# Self‐Healing Starch‐Based Ionogels with Hydroneutral Dipole–Dipole Interactions

**DOI:** 10.1002/advs.202523541

**Published:** 2026-02-10

**Authors:** J. Justin Koh, Jiayi Liu, Xue Qi Koh, Binting Huang, Szu Cheng Lai, Jayce Jian Wei Cheng, Gwendolyn J. H. Lim, Warintorn Thitsartarn, Yong‐Wei Zhang, Zhigen Yu, Chaobin He

**Affiliations:** ^1^ Institute of Materials Research and Engineering (IMRE) Agency for Science, Technology and Research (A*STAR) Singapore Republic of Singapore; ^2^ Department of Materials Science and Engineering National University of Singapore Singapore Republic of Singapore; ^3^ School of Materials Science and Engineering Nanyang Technological University Singapore Republic of Singapore; ^4^ Institute of High Performance Computing (IHPC) Agency for Science, Technology and Research (A*STAR) Singapore Republic of Singapore

**Keywords:** 3D‐printing, dipole–dipole, electronics, e‐skin, self‐healing, water

## Abstract

Transparent ionically conductive self‐healing polymeric materials are essential for enabling many next‐generation technologies in areas including electronics and robotics. However, many of them lose their self‐healing ability when they come into contact with water. Herein, starch‐based, conductive, underwater‐healable and transparent ionogels for soft electronics (SCUTE) are introduced. SCUTEs consist of starch macromolecules that are partially substituted with cyanoethyl groups, and incorporated with hydrophobic ionic liquid tributyl(methyl)ammonium dicyanamide. The aprotic cyanoethyl groups possess a high polarity, thereby capable of forming dipole–dipole interactions stronger than hydrogen bonding of hydroxyl groups. Despite its high polarity, the cyanoethyl groups possess hydroneutral characteristics that only interact weakly with water. This allows dipole–dipole interactions between cyanoethyl groups to be uninterrupted even in the presence of water. More importantly, the synergistic effect between the hydroneutural cyanoethyl dipole–dipole and hydrophilic hydrogen bond led to SCUTEs’ distinct water‐accelerated self‐healing ability. In particular, healing efficiency in stretchability for SCUTE‐20 increased from 37.4% in ambient to 92.0% when exposed to water, for a healing duration of 24 h. To show its potential in soft electronics, SCUTE is demonstrated as electronic skin for robotics control and 3D‐printed aquatic electronics.

## Graphical abstract

This work introduces starch‐based, conductive, underwater‐healable, and transparent ionogels for soft electronics (SCUTEs). Starch macromolecules are partially substituted with cyanoethyl groups and incorporated with hydrophobic ionic liquid. The polar yet hydroneutral cyanoethyl groups facilitate strong dynamic dipole–dipole interactions that remain uninterrupted in the presence of water. Their synergetic effect with hydrophilic hydroxyls leads to SCUTEs’ distinct water‐accelerated self‐healing ability.

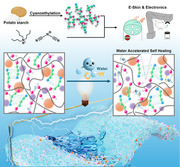

## 1 Introduction

Transparent self‐healable electrically conductive polymeric materials are highly attractive for many next‐generation applications. Electrical conductivity enables the material to perform functions such as charge transport for electrical signalling and redox reactions. On the other hand, self‐healing ability is especially attractive for materials that come under frequent mechanical forces during applications, such as flexible devices and surfaces that are exposed to sporadic mechanical contact/impact of varying magnitude. Therefore, materials that possess the ability to perform both electrical functions and self‐healing have the potential to advance various fields that include electronics [1, 2], robotics [3, 4], biomedical [5, 6], energy harvesting, and energy storage [7, 8, 9]. Coupled with optical transparency, fast‐healing ability and underwater applicability, such self‐healing materials can be exploited in an even wider range of applications.

However, most self‐healing polymeric materials are not intrinsically conductive. As such, conductive fillers, such as carbon and silver nanoparticles, are often incorporated to endow electrical conductivity through their percolation [10]. This typically leads to an opaque composite material, which can be undesirable for many postmodern technologies that require optical transparency, including in the area of optoelectronics [11] and soft robotics [12] fields. Nevertheless, one possible way to achieve transparency, together with conductivity and self‐healing ability, is to incorporate a thin layer of percolated conductive nanomaterials such as graphene or silver nanowires on the surface of the self‐healing polymeric material [11, 13, 14]. An alternative method is to incorporate mobile ions into a polymer matrix to form a homogenous ionically conductive polymeric material [15], for instance, self‐healing hydrogel polymer electrolytes [16, 17, 18]. Furthermore, many self‐healable polymeric materials tend to lose their healing efficiency upon contact with water, thereby limiting their applicability. Therefore, the development of conductive and transparent ionogels that can self‐heal when in contact with water allow higher durability, facile reparations of the ionogels, and their employment in wider range of applications in various environments including aquatic ones.

Self‐healable polymeric ionogels requires dynamic bonds or interactions to facilitate their reformation upon breakage. While dynamic covalent bonds are strong, they typically require elevated temperature to be activated [19, 20]. In contrast, reformation of dynamic secondary interactions can take place autonomously in the operating environment (e.g. ambient). However, most secondary interactions are weaker and are inadequate to hold the polymer structure together as a solid material while having sufficient polymer chain mobility. Among various secondary interactions, hydrogen bonding, being known to be one of the stronger secondary interactions, is the most commonly employed for self‐healing materials [21, 22, 23, 24, 25, 26, 27, 28]. On the other hand, self‐healing materials based primarily on dipole–dipole interactions are rare, as they are typically known to be a weaker form of secondary interactions, as compared to hydrogen bonding. Most reported self‐healable polymeric ionogels employing dipole–dipole interactions are based on fluorinated polymers and ionic liquids, such as plasticized poly(vinylidene fluoride‐co‐hexafluoropropylene) (PVDF‐HFP) and bis(trifluoromethane)sulfonamide [TFSI]‐based ionic liquids [2, 29, 30, 31, 32, 33, 34, 35, 36, 37, 38, 39, 40]. Due to the presence of highly electron‐withdrawing fluorine, fluorinated functional groups can generate large dipoles to facilitate strong dipole–dipole interactions for self‐healing materials. Additionally, unlike typical polar groups that are generally hydrophilic, these fluorinated functional groups are also known to possess the unique hydrophobic characteristics of the C─F bonds [41]. Nevertheless, many of these fluorinated compounds, with trifluoromethyl or difluoromethylene functional groups, are classified as per‐ and polyfluoroalkyl substances (PFAS) that are of environmental and health concerns [42, 43], and are increasingly being regulated to reduce their use by many corporations, governments, and various organizations. To address this, other nonfluorinated highly electron‐withdrawing functional groups can be explored. For instance, the highly electron‐withdrawing cyano (or nitrile) group is known to be able to generate a significant dipole in many small molecules (e.g., acetonitrile). However, despite their high polarity, many of these nitrogen‐containing polar aprotic functional groups were also determined to possess hydroneutral rather than hydrophilic characteristics, as they do not form strong interactions with water [44].

**FIGURE 1:**
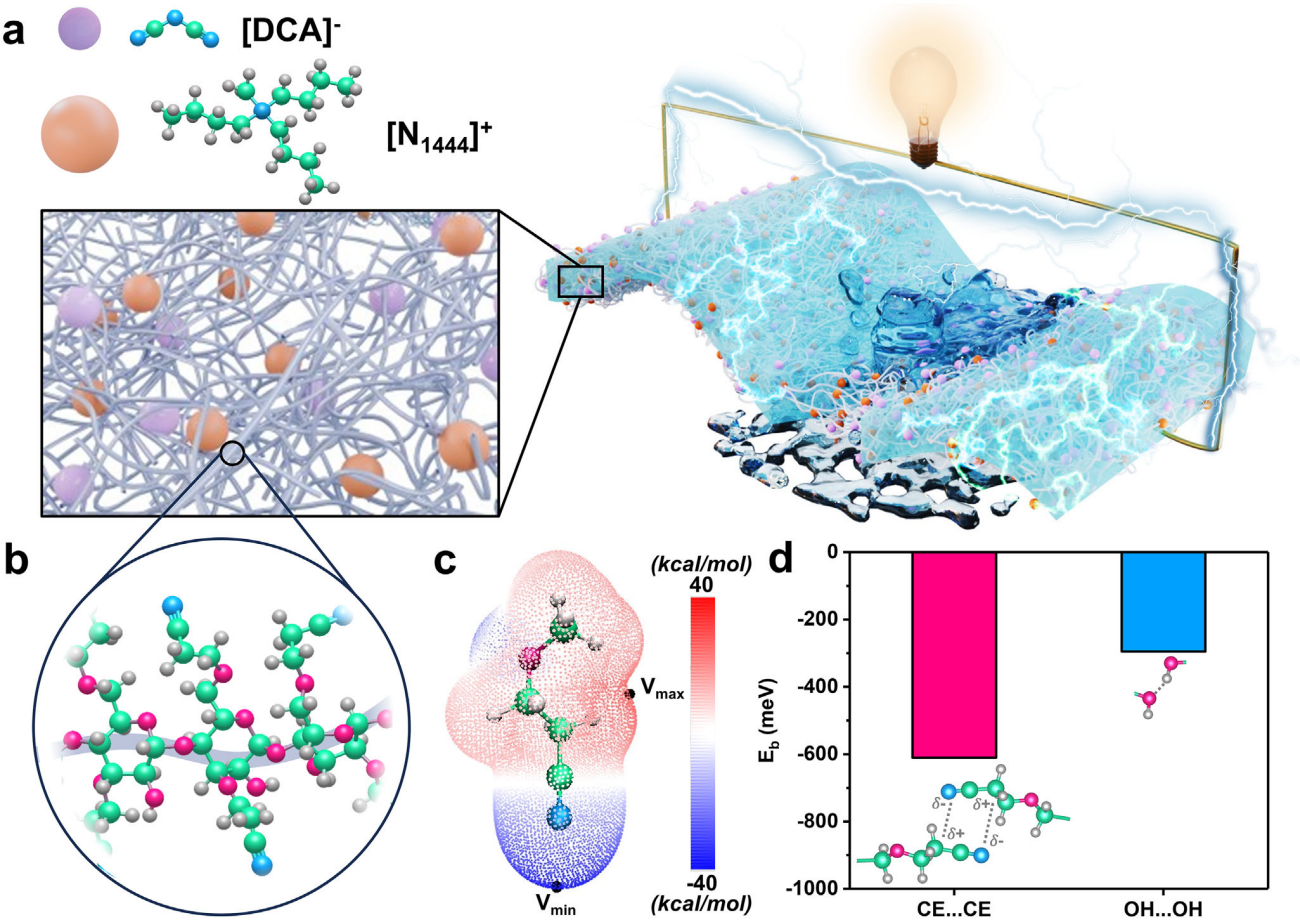
Concept and design of SCUTE. (a) Schematic illustrating the concept and composition of SCUTE. (b) Schematic illustrating starch partially substituted with cyanoethyl group (CEStarch) in SCUTE. (c) Electrostatic potential distribution of CE group model. (d) Binding energy of dipole–dipole interactions between two CE groups and hydrogen bond interactions between two OH group of CEStarch calculated by DFT simulations. Atomic labelling: oxygen‐pink, nitrogen‐blue, carbon‐green, hydrogen‐light grey.

Herein, a self‐healable starch‐based ionogel has been developed, using partially substituted cyanoethyl starch (CEStarch) and tributyl(methyl)ammonium dicyanamide ([N_1444_][DCA]). Unlike most self‐healing polymers that typically introduce one type of dynamic interaction [45], the partial substitution of linear amylose and branched amylopectin allows them to possess both cyanoethyl and hydroxyl functional groups, thereby capable of forming dynamic dipole–dipole interactions and hydrogen bonding to facilitate self‐healing ability. On the other hand, [N_1444_][DCA] is a hydrophobic ionic liquid that serves simultaneously as the charge carrier and plasticizer to enhance chain mobility of CEStarch. As such, a starch‐based, conductive, underwater‐healable, and transparent ionogel for soft electronics (SCUTE) was developed, as illustrated in Figure 1a. While SCUTE possess autonomous self‐healing ability, its healing efficiency can also be effectively accelerated upon contact with water, attributed to the synergy between the hydrogen bonding of the hydrophilic hydroxyl groups and the dipole–dipole interactions of the hydroneutral cyanoethyl groups. To show its practical applicability and versatility, SCUTE is also 3D‐printed, and demonstrated in various applications such as electronic skins (e‐skins) for robotics control and flexible aquatic electronics in this study.

## 2 Results

### 2.1 Synthesis and Fabrication of SCUTE

CEStarch is synthesized from water‐soluble potato starch in alkaline aqueous solution. Unlike its linear cellulose analog with extended β‐1,4‐glycosidic linkages, starch contains two macromolecular components, linear amylose and branched amylopectin chain, both with bent α‐1,4‐glycosidic linkages, and also branching with α‐1,6‐glycosidic linkages for amylopectin. The bent conformation and branching structure provide starch with lower interchain interactions, which are beneficial for its chain mobility and facilitate water solubility. Upon substitution with cyanoethyl (CE) functional group via reaction with acrylonitrile in alkaline aqueous solution, CEStarch gradually precipitates and clumps together. The purified CEStarch obtained is insoluble in water, indicating a reduction in hydrophilicity in comparison with the pristine water‐soluble potato starch. Following, [N_1444_][DCA] is incorporated into CEStarch, which performs a dual role as the charge carriers for electrical conductivity, as well as a plasticizer for CEStarch. The resultant homogeneous ionogel is denoted as SCUTE, analogous to the shell of amphibious turtles.

As a result of partial substitution, CEStarch contains both unsubstituted hydroxyl groups and substituted CE groups (Figure 1b). To elucidate the polarity of the CE group, density functional theory (DFT) calculations were conducted to show the charge distribution of a CE group model. The electrostatic potential (ESP) distribution map presented in Figure 1c shows that, indeed, electrons are highly localized around the nitrogen atom, resulting in an ESP minimum of −38.81 kcal mol^−1^ at the tip of the nitrogen atom. On the other hand, the ESP maximum of 26.24 kcal mol^−1^ lies around the hydrogen atom bonded to the second carbon atom from the nitrogen, which is also between two electronegative ether and cyano functional groups. DFT calculations were also conducted to calculate the binding energies (E_b_) between two CEStarch molecular models (Figure S1). Indeed, the two polar CE groups are capable of forming very strong dipole–dipole interactions, having a binding energy of around −610 meV. This is in fact more than two times in magnitude, as compared to −295 meV of the hydrogen bonding formed between the OH groups of CEStarch (Figure 1d). This result indicates the capability of CE groups in forming dipole–dipole interactions stronger than the hydrogen bonding by the OH groups.

### 2.2 Structure and Physical Properties of SCUTE

**FIGURE 2:**
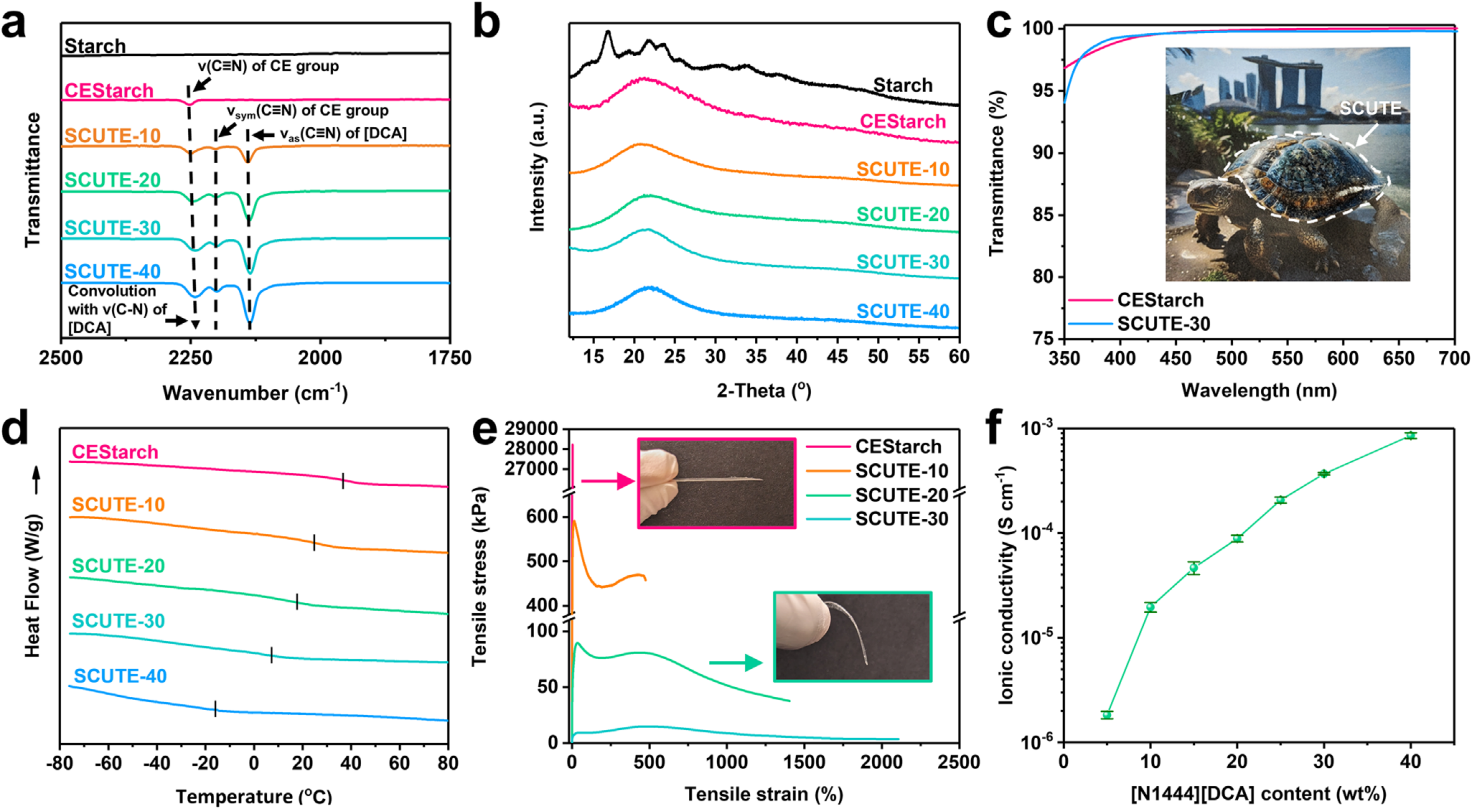
Structure and physical properties of SCUTEs. (a) ATR‐FTIR spectroscopy of starch, CEStarch and SCUTEs. (b) X‐ray diffractogram of starch, CEStarch, and SCUTEs. (c) Ultraviolet–visible (UV–vis) spectroscopy measuring transmittance of 300 µm thick CEStarch and SCUTE‐30 as a representative of SCUTEs, between the wavelength of 350 to 700 nm. Inset shows a turtle picture with a 400 µm thick SCUTE‐20 placed on top of the turtle shell, highlighting the transparency of SCUTEs (d) Differential scanning calorimetry (DSC) measurement of CEStarch and SCUTEs heating curve. Line on curve indicates the glass transition temperature. (e) Tensile stress‐strain curves of CEStarch and SCUTEs. Insets show the rigid‐to‐flexible transition from CEStarch to SCUTE‐20. f Ionic conductivity of SCUTEs with error bars as a function of [N_1444_][DCA] ionic liquid content measured using electrochemical impedance spectroscopy. The error bars represent the standard deviation.

The successful grafting of CE group onto starch was confirmed by nuclear magnetic resonance (NMR) spectroscopy (Figure S2) and attenuated total reflectance—Fourier transform infrared spectroscopy (ATR‐FTIR) presented in Figure 2a and Figure S3. The appearance of the characteristic peak at wavenumber of around 2250 cm^−1^ can be attributed to C≡N stretching vibration ν(C≡N) of the grafted CE group [46, 47]. The synthesized CEStarch has a degree of substitution (DS) of around 1.79, as determined by elemental analysis (Table S1). This suggests that the as‐synthesized CEStarch possesses around 1.2 hydroxyl groups (OH) per anhydroglucose unit (AGU), assuming a maximum of DS of 3. The successful incorporation of [N_1444_][DCA] to form SCUTE‐X (X is the wt% of [N_1444_][DCA]), can be observed from the appearance of the strong FTIR absorption peak at wavenumber around 2130 cm^−1^ arising from the asymmetric C≡N stretching vibrations ν_as_(C≡N) of the [DCA] anion [48, 49]. The distinct symmetric stretching of C≡N ν_sym_(C≡N) of [DCA] can also be observed at wavenumber around 2190 cm^−1^ for SCUTEs, having increasing absorption intensity with increasing [N_1444_][DCA] content. The apparent blue shift of the FTIR characteristic peak around wavenumber 2250 cm^−1^ is due to the convolution with the combination band of the N–C stretching modes of [DCA], both symmetric ν_sym_(C–N) and antisymmetric ν_as_(C–N), at wavenumber around 2225 cm^−1^.

Pristine potato starch has a semi‐crystalline structure, as shown in the X‐ray diffractogram presented in Figure 2b. The partial and random substitution of the spatially bulky CE groups onto starch macromolecules disrupts the regularity of starch marcromolecules, thereby hindering the formation of ordered crystal [50], leading to CEStarch having an amorphous structure, as well as the [N_1444_][DCA]‐infused SCUTEs. More importantly, the CEStarch and [N_1444_][DCA] possess high miscibility, leading to a homogenous ionogel. Such high compatibility between CEStarch and [N_1444_][DCA] can be attributed to the multiple interacting sites between the three components, including dipole‐ion interactions. Indeed, DFT calculations show negative binding energies of significant magnitude between the CEStarch and [N_1444_][DCA] at multiple molecular orientations (Figure S4), indicating strong interactions and high compatibility between them.

The homogeneity of SCUTEs is also reflected in its optical properties. CEStarch and SCUTE ionogels show high optical transparency of more than 95% transmittance in the visible light range between 380 and 700 nm (Figure 2c and Figure S5). In addition, their high compatibility allows [N_1444_][DCA] to serve as an effective plasticizer to CEStarch. Differential scanning calorimetry of (DSC) CEStarch and SCUTEs shows decreasing of CEStarch glass transition temperature with increasing [N_1444_][DCA] content, from 39.7°C of CEStarch to 28.3°C, 18.7°C, 6.8°C, and −17.9°C of SCUTE‐10 to SCUTE‐40, respectively (Figure 2d). The lower T_g_ of SCUTEs also indicates a higher chain mobility due to reduced interchain interaction. As such, the plasticization effect can be observed from their mechanical properties (Figure 2e). Pristine CEStarch is a rigid and brittle glassy material with a tensile modulus of 1.0 ± 0.02 GPa, tensile strength of 21.8 ± 2.2 MPa, and elongation at break of 4.5 ± 0.6%. With increasing [N_1444_][DCA] content, SCUTE ionogels exhibit increasing flexibility and ductility. Significant stretchability enhancements can be observed in the increase in elongation at break, from 447 ± 13.5% to 1414 ± 62% and 2104.2 ± 254.1% for SCUTE‐10, SCUTE‐20, and SCUTE‐30, respectively. On the other hand, tensile modulus reduced from 51.1 ± 5.7 MPa, to 2.5 ± 0.8 MPa and 0.30 ± 0.02 MPa, while tensile strength reduced from 600.4 ± 23.5 kPa to 87.7 ± 3.6 kPa and 11.4 ± 0.2 kPa for SCUTE‐10, SCUTE‐20, and SCUTE‐30, respectively. In particular, SCUTE‐20 and SCUTE‐30 are structurally stable viscoelastic materials that can be easily handled (Figures S6–S8). Furthermore, having [N_1444_][DCA] serving concurrently as the charge carriers, electrical conductivity also increases with increasing [N_1444_][DCA] content. As shown in Figure 2f, the ionic conductivity increases from the order magnitude of 10^−6^ S cm^−1^ for SCUTE‐5 to around 8.5 × 10^−4^ S cm^−1^ for SCUTE‐40, as measured by the electrochemical impedance spectroscopy technique (Figure S9). Besides higher charge carrier concentration, higher charge carrier mobility due to increased CEStarch chain mobility could possibly also contribute to higher ionic conductivity. In general, SCUTEs, in particular SCUTE‐20 and SCUTE‐30 possess comparable physical properties to many of the self‐healing polymeric materials in literature (Table S2). Furthermore, SCUTEs are also highly recyclable given the noncovalent nature of the polymer network. As such, green solvents (water and acetone) can be used to reprocess used SCUTEs (Figure S10). Such a solvent‐based recycling method is demonstrated to be effective in the preservation of SCUTEs’ physical properties (Figure S11).

### 2.3 Self‐Healing Ability of SCUTE

**FIGURE 3:**
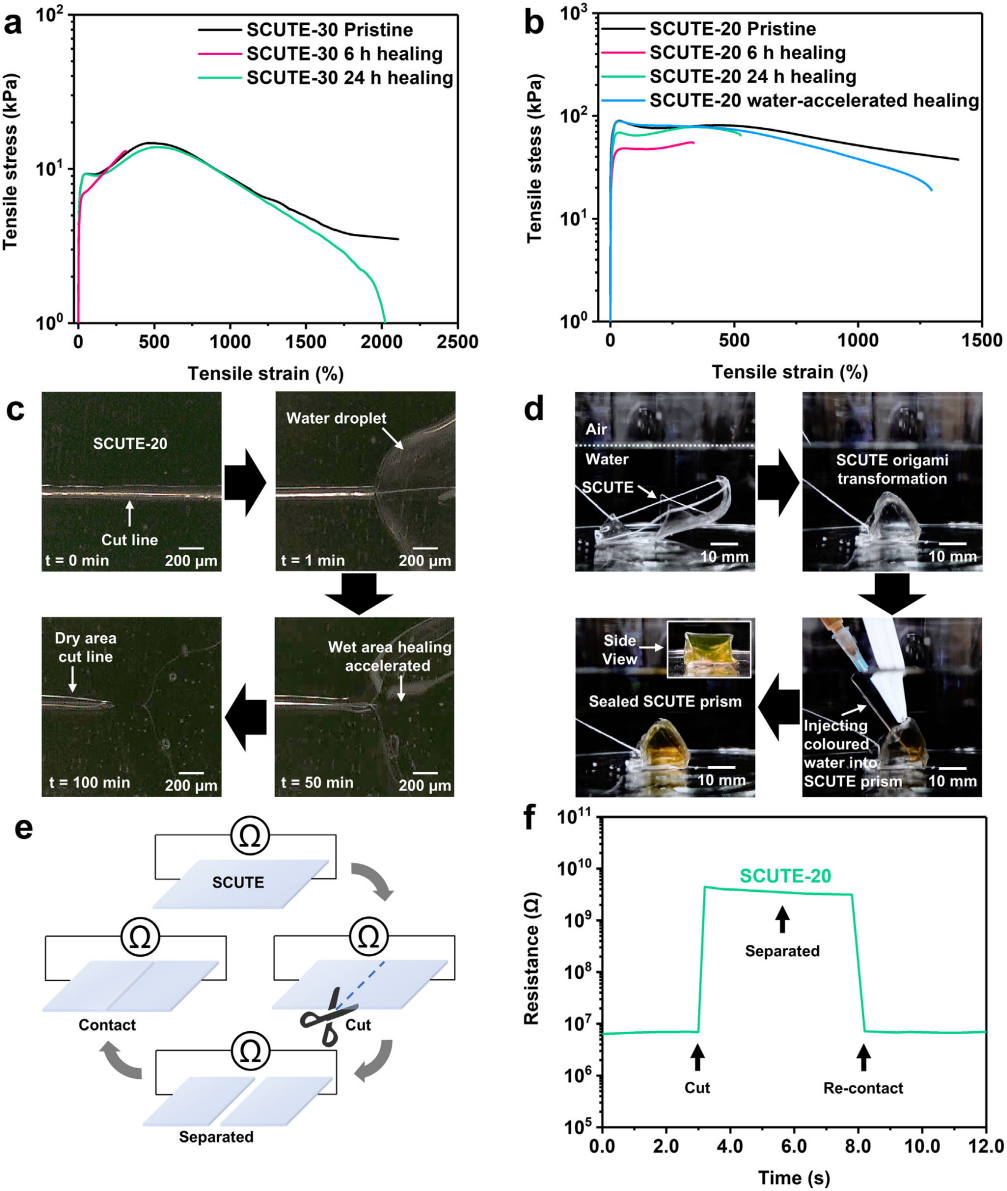
Self‐healing abilities of SCUTEs. (a) Tensile stress‐strain curves showing mechanical self‐healing of SCUTE‐30 in ambient environment. (b) Tensile stress‐strain curves showing mechanical self‐healing of SCUTE‐20 in the ambient environment and water‐induced accelerated self‐healing. (c) Microscopic images showing the effect of water‐accelerated self‐healing along a cut line with dry and wetted regions. (Video S1). (d) Demonstration of SCUTE‐20 underwater self‐healing ability. A two‐dimensional SCUTE‐20 sheet undergoing origami transformation and self‐healing into a prism‐shaped capsule, followed by injection with coloured water into the sealed capsule (Video S2). (e) Schematic diagram illustrating the electrical self‐healing demonstration of SCUTE. f Resistance measurement of an SCUTE‐20 sheet as a function of time, as the material undergoes a cutting, separation, and electrical healing process.

The mechanical self‐healing abilities of the ionogels were analysed with consideration of both their yield strength and ductility/stretchability. Indeed, with adequate chain mobility provided by sufficient amount of [N_1444_][DCA] as the plasticizer, along with the dynamic interactions, SCUTE‐30 exhibits excellent autonomous self‐healing ability (Figure 3a). In 6 h, SCUTE‐30's yield strength recovers by 88.9%, albeit only a 14.8% recovery in stretchability. However, SCUTE‐30 shows high recovery efficiency of up to 94.2% recovery in yield strength and 97.3% recovery in stretchability after 24 h of autonomous healing in ambient conditions. On the other hand, SCUTE‐20 contains less plasticizer, and hence, lower CEStarch chain mobility, as evident from its higher T_g_ as compared to SCUTE‐30 (Figure 2d). This translates into lower healing efficiency, such that SCUTE‐20 only shows 53.9% and 77.0% recovery in yield strength, 23.9% and 37.4% recovery in stretchability after 6 h and 24 h healing time, respectively in ambient environment (Figure 3b). This shows that even though healing has been initiated and taken place autonomously in the ambient environment, the slow kinetics have led to incomplete healing even after 24 h. However, if the damaged pieces of SCUTE‐20 were to come into contact with water, healing can take place very quickly. Using a cut SCUTE‐20 sample, water was dripped on the cut region and allowed self‐healing (and drying) to take place in the ambient environment for 24 h. The water‐accelerated healing allows the healed SCUTE‐20 to achieve 99.1% recovery in yield strength and 92.0% recovery in elongation at break.

To better visualize the water‐accelerated self‐healing ability, a piece of SCUTE‐20 was cut using a sharp blade, and water was dripped on a segment of the cut line. The microscopic images in Figure 3c shows the healing process of the dry and wetted regions of the cut line on SCUTE‐20 (Video S1). Indeed, the cut line in the wetted region disappeared in less than 50 min while the cut line in the dry region remains observable. Such water‐accelerated rapid self‐healing characteristic can also be advantageous at low temperatures where autonomous self‐healing kinetics are retarded beyond meaningful levels, especially if the temperature is below the T_g_ of the material (Figure S12) [51, 52]. Furthermore, the mechanical properties of SCUTE‐20 remain comparable to the its pristine state even after multiple cycles of water‐accelerated self‐healing (Figure S13). To further demonstrate the self‐healing ability of SCUTE in aquatic environment, while maintaining its structural integrity, a two‐dimensional SCUTE‐20 sheet shown in Figure 3d is made to undergo origami transformation and self‐healing in aqueous medium to form a sealed triangular prism capsule (Video S2). Briefly, a SCUTE‐20 sheet threaded with cotton threads at its vertices was submerged underwater. Tightening of the threads enables its transformation from a two‐dimensional sheet into a three‐dimensional triangular prism capsule. The sealing of the capsule is then facilitated by water‐accelerated self‐healing. Subsequent injection of coloured (yellow) water into the core of the seal prism capsule demonstrates the sealing integrity of the prism through effective underwater self‐healing, and the high transparency of SCUTEs.

Apart from mechanical healing, it is also necessary for SCUTEs to possess electrical self‐healing abilities, especially for electronics applications. The self‐healing property of the material was demonstrated by measuring the electrical resistance across SCUTE sheets as illustrated in Figure 3e, whereby the SCUTE sheets would undergo physical incision, separation, and subsequent re‐contact. As shown in Figure 3f, the measured resistance sharply increased by several orders of magnitude upon the incision and separation of the cut SCUTE‐20 sheets, indicating the disruption of the conductive pathways. Indeed, electrical conductivity recovers instantaneously with the measured resistance dropping back to its original value upon re‐contact, confirming the material's ability to restore its electrical integrity. SCUTE‐30 exhibits similar electrical healing behaviour (Figure S14).

### 2.4 SCUTE Interaction with Water

This section discusses the interaction between SCUTE and water to elucidate the origins of SCUTE's distinct self‐healability in the presence of water. Firstly, [N_1444_][DCA] is a hydrophobic ionic liquid that exhibits immiscibility with water. This characteristic changes drastically when the cation is switched to 1‐ethyl‐3‐methylimidazolium dicyanamide ([Emim][DCA]), which suggests that its hydrophobicity is contributed significantly by the alkyl chains of the [N_1444_] cation, as shown in Figure S15. On the other hand, while CEStarch is derived from water‐soluble starch, its reduced hydrophilicity can be inferred from its precipitation from the aqueous solvent upon substitution with CE groups, and its subsequent insolubility in water. However, the partially substituted CEStarch retains slightly less than half of its original hydrophilic OH groups, around 1.2 per AGU. While the OH groups can contribute to the strong dynamic interchain interactions through hydrogen bonding, they are also known to have high affinity with water. As such, SCUTEs exhibit a mild water‐absorbing characteristic, having around 15 wt.% more absorbed water content upon contact with excess water as compared to its dry ambient state (Figure S16). The small amount of absorbed water can then serve as temporary plasticizer by “unlocking” the interchain hydrogen bonding. This would significantly increase the chain mobility of CEStarch in SCUTE, leading to the accelerated self‐healing ability.

**FIGURE 4:**
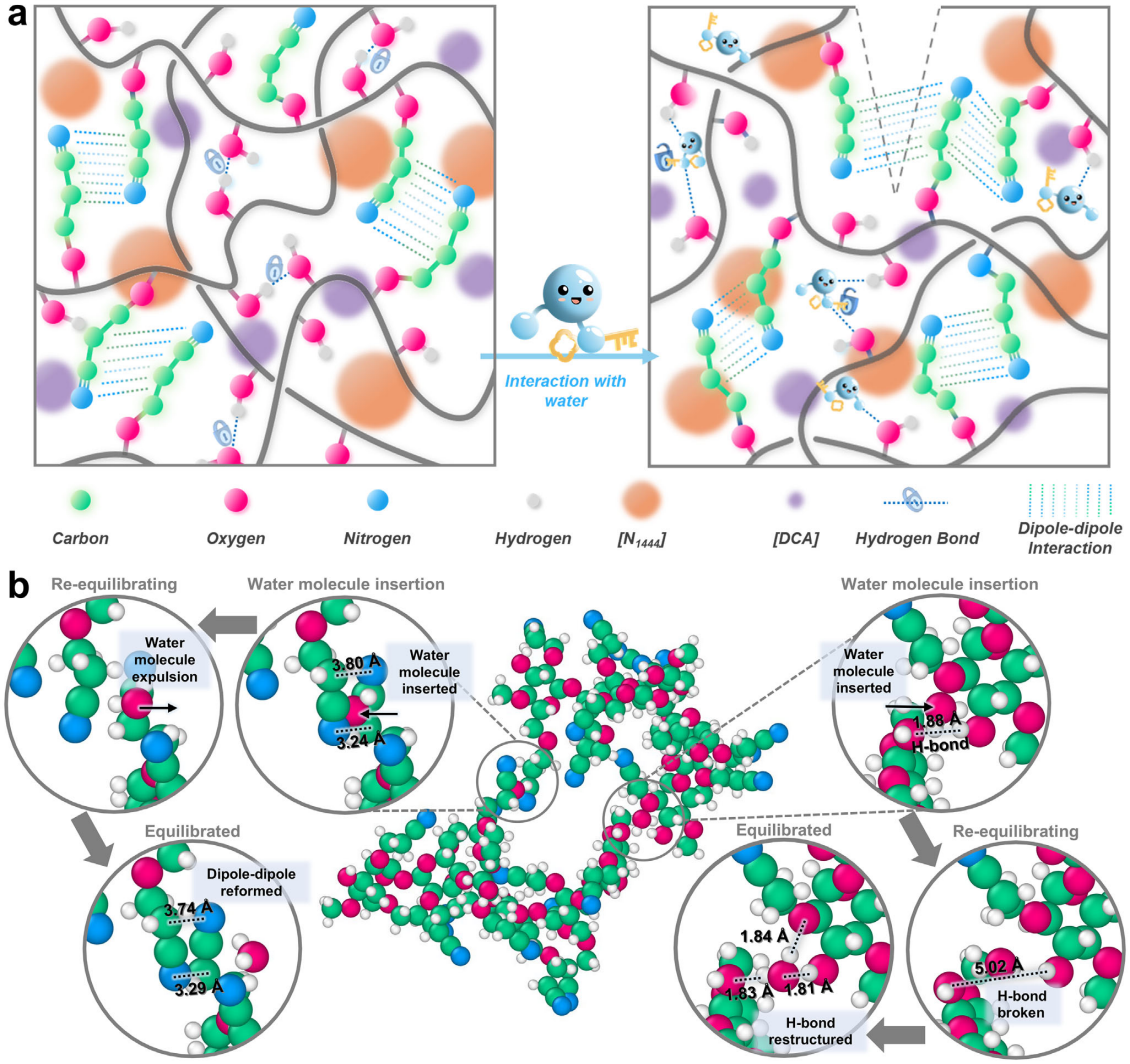
SCUTE‐Water interactions. (a) Schematic diagram illustrating the synergetic mechanism of CE···CE dipole–dipole interactions and OH···OH hydrogen bonding when SCUTE interacts with water, leading to its underwater and water‐accelerated self‐healing ability. (b) Re‐equilibration DFT simulation of water molecules inserted between (left) pre‐equilibrated CE···CE dipole–dipole interactions and between (right) pre‐equilibrated OH···OH hydrogen bond interactions. Atomic labelling: oxygen‐pink, nitrogen‐blue, carbon‐green, hydrogen‐light grey (Video S3).

On the other hand, strong dipole–dipole interchain interactions between the highly polar CE groups must be preserved for SCUTE to maintain its structural integrity. Dry SCUTE‐20 exhibits a high yield stress in the 10^5^ Pa order of magnitude as determined using the rheological stress ramp method [53]. Albeit some reduction in yield stress as compared to its dry state, wet SCUTE‐20 possesses a yield stress in the 10^4^ Pa order of magnitude, measured immediately after removal from aqueous environment (Figure S17a–c). It is noteworthy that the yield stress level is high when compared to many other gel materials. The enhanced chain mobility can be inferred from the lower viscosity of SCUTEs in their wet state across the entire range of shear stress levels measured, as compared to their dry state (Figure S17d,e). Indeed, according to previous studies, these cyano‐based functional groups were determined to be hydroneutral despite having a high polarity, and hence interact weakly with water molecules [44, 54]. This would allow minimal disruption to the strong dipole–dipole interactions between the CE groups even with the presence of absorbed water content in SCUTEs. Such a dual interaction mechanism resembles that of poly(methyl methacrylate/n‐butyl acrylate) [P(MMA/nBA)] copolymer, which relies on the synergetic hydrogen bonding and van der Waals interactions in the presence of water for accelerated self‐healability [55]. Similarly, the synergetic effect between the dynamic hydrogen bonding and dipole–dipole interactions in facilitating SCUTEs’ accelerated self‐healing ability in the presence of water is illustrated in Figure 4a.

To confirm the hydroneutrality of the CE groups, DFT simulations were performed. Water molecules were inserted intentionally between two pre‐equilibrated interacting CE groups (dipole–dipole), and between two pre‐equilibrated interacting OH groups (hydrogen bonding) of separate CEStarch macromolecules (Figure 4b) (Video S3). Following the water molecules insertion, a re‐equilibration process proceeds to elucidate how these interacting functional groups behave in the presence of water. For the CE groups, the water molecule inserted in between them were expelled during the re‐equilibration process. This leads to the atomic distances between the electron‐rich nitrogen atoms and the electron‐deficient carbon atoms reverting back to the pre‐equilibrated state upon expulsion of the inserted water molecule, at around 3.2–3.8 Å, indicating the reformation of the dipole–dipole interaction between the CE groups. More importantly, the results confirm the hydronetural characteristics of the CE···CE dipole–dipole interactions, and their ability to retain these interactions even in the presence of water. In contrast, the inserted water molecule between interacting OH groups led to the breakage of their hydrogen bond interactions. This is indicated by the distancing of the originally interacting OH···OH from 1.88 Å in the pre‐equilibrated state to 3.50 Å in the re‐equilibrated state after the insertion of water. Given the short‐range nature of hydrogen bond interactions, the acceptable cutoff distance is typically below 2.6 Å [56, 57]. The final equilibrated state shows the inserted water molecule separating the two OH groups through the formation of hydrogen bonding with each OH group individually, with bond distances of around 1.8 Å, thereby indicating the hydrophilic characteristic of the OH groups. Combining both the hydroneutral and hydrophilic characteristics of CE and OH groups, respectively, explains their synergetic effect, leading to SCUTEs’ distinct self‐healing behaviour in the presence of water.

A quick comparison with other ionogels that employ the dipole strategy shows SCUTEs’ uniqueness (Figure S18) [2, 30, 31, 35, 37, 39, 58, 59, 60]. Most of these ionogels that exhibit self‐healability in the presence of water uses fluorinated synthetic compounds, exploiting the highly electron‐withdrawing fluorine group to generate a large dipole along with fluorocarbons’ unique hydrophobicity [41]. This allows SCUTEs to stand out in the sustainability aspect, offering a bio‐based, per‐ and polyfluoroalkyl (PFAS)‐free and recyclable alternative, while achieving comparable mechanical, electrical, and optical performance. Interestingly, these PFAS‐based self‐healable ionogels employing hydrophobic polar fluorocarbon groups also experience a retarded healing ability in the presence of water, as compared to their healing ability in the ambient environment (Table S3). This highlights SCUTEs’ unique strategy of combining of hydroneutral dipole–dipole and hydrophilic hydrogen bonding groups that facilitated its water‐accelerated self‐healability.

### 2.5 SCUTE for Soft Electronics Applications

**FIGURE 5:**
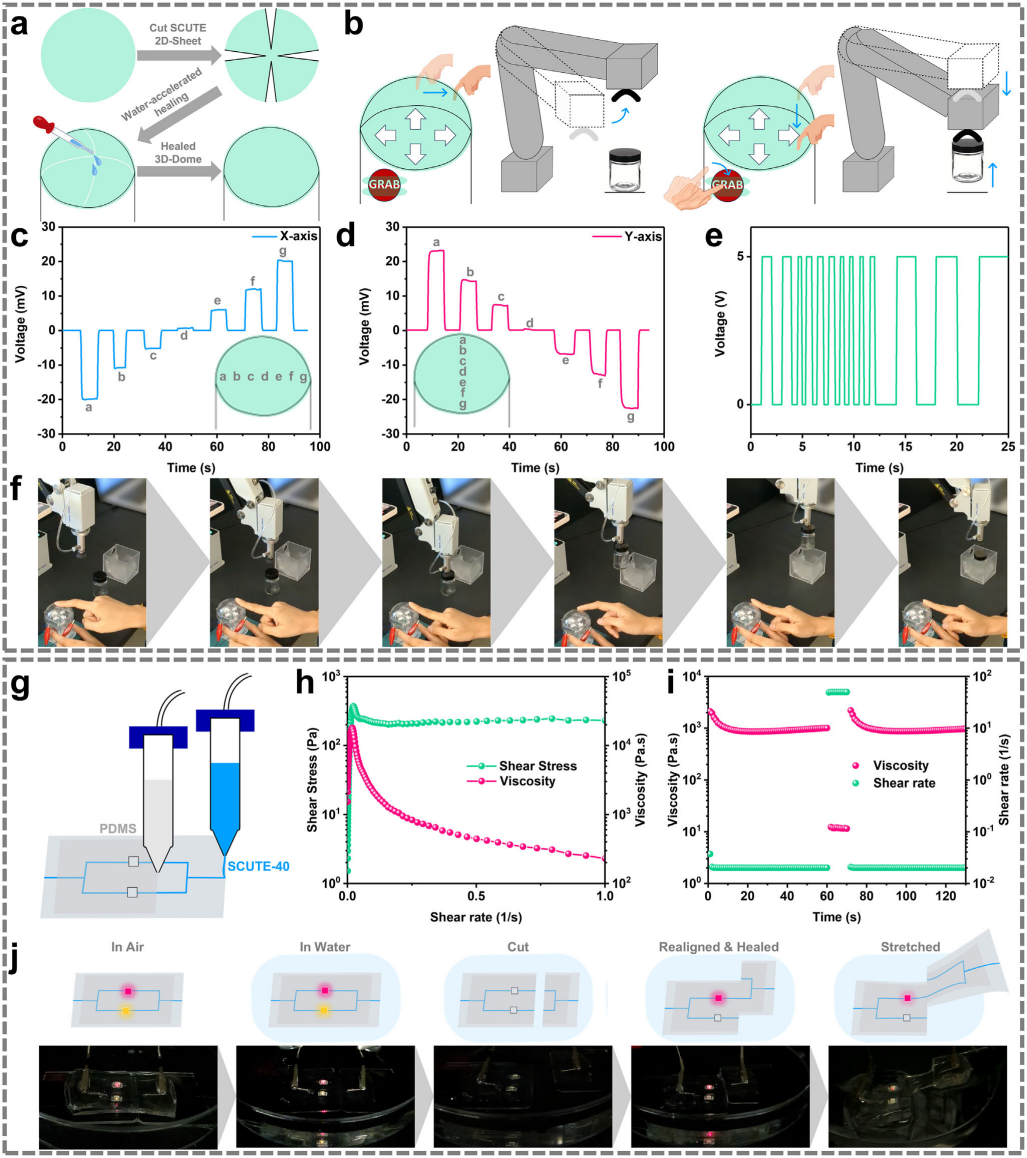
SCUTEs in soft electronics applications. (a–f) SCUTE‐20 e‐skin for robotic arm control. (a) Schematic illustration of the fabrication process of dome‐shape flexible SCUTE‐20 e‐skin through slice removal and water‐accelerated self‐healing. (b) Schematic illustration of SCUTE‐20 e‐skin serving as the human‐machine interface for the robotic arm control. (c and d) Voltage readings of lock‐in amplifiers when positions were touched across (c) x‐axis and (d) y‐axis, separately. (e) Digital signal when “GRAB” button was touched (5 V) or released (0 V). (f) Images showing the demonstration of employing SCUTE e‐skin as the human‐machine interface for the robotic arm control (Video S4). (g–j) 3D‐printed aquatic electronics. (g) Schematic diagram illustrating the 3D‐printing of a circuit board prototype using SCUTE‐40 ink for conductive track and a PDMS ink for the substrate. (h and i) Rheological behaviour of SCUTE‐40 ink. (h) Stress ramp measurement with shear stress and viscosity as a function of shear rate. (i) Step‐shear‐rate measurement, with viscosity a function of three shear rate steps (low, high, low). (j) Demonstration of the 3D‐printed circuit board undergoing damaging and healing process in aquatic environment (Video S5).

This section demonstrates SCUTE ionogels employment in electronics applications, as well as showcasing its water‐accelerated and underwater self‐healing ability. In the first example, SCUTE‐20 is employed as e‐skins for robotics control. Figure 5a illustrates the fabrication process of a 3D dome‐shaped e‐skin from a circular 2D SCUTE‐20 sheet, by cutting out several slices followed by water‐accelerated healing of the sliced edges. The healed SCUTE‐20 dome shows no observable “cut” lines and conforms perfectly onto a rigid dome substrate. A surface‐capacitive system with two lock‐in amplifiers connected perpendicularly across SCUTE‐20 e‐skin was set up (Figure S19), to measure the small impedance change of the sensor induced by the body capacitance when touched at various positions. The adequate ionic conductivity of SCUTE‐20 helps to facilitate the quick detection of the body capacitance and the real‐time touch positions, enabling fast responses (>30 Hz) necessary to control the lateral and vertical movement of a robotic arm as illustrated in Figure 5b. Additionally, a digital e‐skin was formed by two rectangular strips of SCUTE‐20 aligned in parallel and separated by a 2 mm gap to serve as digital control for the “grabbing” and “releasing” motion of the robotic arm. Icons serving as user interface guides, such as arrows and “GRAB,” were pasted under the e‐skins, respectively, exploiting the optical transparency of the transparent e‐skin. Figure 5c,d shows the voltage readings of the two lock‐in amplifiers when locations across the x‐axis and y‐axis of the dome‐shaped SCUTE‐20 e‐skin were touched, respectively. Figure S20 shows the simultaneous voltage readings of both the lock‐in amplifiers when different locations of the SCUTE‐20 e‐skin were touched. Figure 5e shows the digital signal of the dual‐strip e‐skin when the “GRAB button” was touched (5 V) and released (0 V). To demonstrate the control of the robotic arm using the SCUTE‐20 e‐skin, snapshots presented in Figure 5f shows the moving of a glass bottle into a cubic container by the robotic arm, controlled using the SCUTE‐20 e‐skin (Video S4).

The water‐accelerated and underwater healability of SCUTEs can also be advantageous for aquatic electronics. Apart from self‐healing ability, it is also important to prevent the leeching of the charge‐carrying ions in the aquatic environment, especially for extended durations. For this purpose, a protective coating made of underwater self‐healable polydimethylsiloxane‐based material with urea linkages (PDMSurea) developed in our previous work [61], can be applied. A thin layer of the PDMSurea coating can preserve SCUTEs’ mechanical and electrical properties in aquatic environment for extended periods (Figure S21). Therefore, in the second example, an aquatic self‐healing printed circuit board (PCB) was fabricated using 3D‐printing method (Figure 5g). The printed PCB consists of two LED light bulbs (red and yellow) connected by SCUTE conductive tracks that are embedded within the non‐conductive PDMSurea. For higher ionic conductivity, SCUTE‐40 was employed as the conductive tracks. Both the SCUTE‐40 and PDMSurea inks were formulated to achieve suitable rheological behaviour for direct‐ink‐writing. They both possess gel‐like behaviour with a yield stress of around 2 × 10^2^ Pa, and a shear‐thinning behaviour upon yielding (Figure 5h) (Figure S22a). More importantly, the SCUTE‐40 ink exhibits a non‐thixotropic behaviour, and self‐heals instantaneously when transiting from a shearing state to a resting state. This can be observed from the step‐shear‐rate viscosity measurement shown Figure 5i, whereby the viscosity of the SCUTE‐40 ink recovers almost instantaneously when the shear rate is switched from high to low. Such rheological behaviour is beneficial for preventing the printed conductive tracks from spreading upon being extruded. On the other hand, the PDMSurea ink is thixotropic (Figure S22b), whereby mild spreading would help to ensure adjacent printed lines impinge on one another to facilitate the printing of a smooth and continuous surface. Demonstration of the underwater self‐healability of the printed PCB is shown and illustrated in Figure 5j. At the start, both the embedded red and yellow LED bulbs can be lighted up in an ambient and underwater environment. The PCB was then cut into two pieces and realigned before re‐contacting for healing to take place underwater for 1 h. The realignment was done in a staggered manner such that only the red LED bulb was placed within a closed circuit of the conductive tracks. After an hour, the underwater healed PCB only lights up the red LED bulb, indicating that the PCB is electrically healed. Furthermore, the healed PCB can also be mechanically stretched with the red LED bulb continuously being lighted, an indication that mechanical healing has also taken place (Video S5).

## 3 Discussion

In summary, a starch‐based, conductive, underwater‐healable and transparent ionogel for soft electronics (SCUTE) is introduced. SCUTE consist of partially substituted starch‐based macromolecular matrix, CEStarch, and a hydrophobic ionic liquid, [N_1444_][DCA]. [N_1444_][DCA] which performs the role of plasticizer to enhance CEStarch's chain mobility, as well as mobile charge carrier for ionic conductivity. Their excellent compatibility results in the formation of homogeneous SCUTE ionogels.

More importantly, the partial substitution of starch allows CEStarch to possess of both grafted CE and the remaining OH functional groups. The grafted CE groups are capable of forming strong dynamic secondary interactions in the form of dipole–dipole interactions. Based on DFT calculations, the dipole–dipole interactions formed by CE can be stronger than hydrogen bonding between OH groups. Nevertheless, it is the synergy between these two secondary interactions that facilitated the water‐accelerated and underwater self‐healing ability of SCUTE. In brief, the hydroneutral characteristics of the CE groups only interacts weakly with water, allowing SCUTE to maintain its dipole–dipole interactions in aqueous environment, and therefore its structural integrity and gel‐like structure. On the other hand, the disruption of OH groups’ hydrogen bonding by the mild absorbed water content, provided additional plasticizing effect and higher chain mobility of CEStarch, which in turn leads to faster self‐healing kinetics taking place. SCUTE was also demonstrated for its versatility in practical soft electronics applications. Firstly, SCUTE‐20 was cut and healed quickly with water to form a 3D dome‐shaped e‐skin. The SCUTE e‐skin was demonstrated to control a robotic arm. In addition, a self‐healable PCB was 3D‐printed with SCUTE‐40 as the conductive track and an underwater self‐healing PDMS as the substrate. The aquatic flexible PCB exhibits excellent performance and self‐healing ability even in an aqueous environment.

While some self‐healing polymeric materials or ionogels have employed the dipole strategy to maintain the self‐healing ability in the presence of water, they typically consist of the highly electron‐withdrawing fluorine group to generate a dipole. From a sustainability perspective, SCUTE offers a PFAS‐free, recyclable, and bio‐based alternative. Furthermore, PFAS‐based self‐healable ionogels employing fluorinated polar groups often experience a retarded healing kinetics in the presence of water, whereas SCUTE exhibits water‐accelerated self‐healability through the synergy of hydroneutral and hydrophilic dynamic secondary interactions. The true potential of SCUTE lies beyond electronics, and could possibly be an enabler for next‐generation technologies in a various fields including, biomedical and energy storage, as a sustainable and long‐lasting material.

## 4 Experimental Section

### 4.1 Materials

Water‐soluble potato starch (≤100%, Sigma‐Aldrich), acrylonitrile (≥99%, Sigma‐Aldrich), sodium hydroxide (NaOH, ≥97%, pellets), and N,N‐dimethylformamide (DMF, 99.8%, Thermo Scientific), poly(dimethylsiloxane), bis(3‐aminopropyl) terminated (PDMS, Mn: 2500 and 27000 g mol^−1^, Sigma‐Aldrich), N‐Tributyl‐N‐methylammonium dicyanamide ([N_1444_][DCA], 99.5%, Solvionic), 1‐ethyl‐3‐methylimidazolium dicyanamide ([Emim][DCA]) (99.5%, Solvionic), 1‐ethyl‐3‐methylimidazolium methanesulfonate ([Emim][MeSO_3_]) >95%, Sigma Aldrich) isophorone diisocyanate (98%, mixture of isomers, Sigma‐Aldrich), anhydrous tetrahydrofuran (>99.9%, Sigma‐Aldrich), acetonitrile (99.8%, Sigma‐Aldrich), 2‐propanol (99.7%, QRec) were used directly upon purchased. Millipore water (18 MΩ cm at 25°C) was used throughout the entire study.

### 4.2 Synthesis of CEStarch and Fabrication of SCUTE

A 20% (w/w) NaOH solution was prepared by dissolving 10 g of NaOH in 40 mL of deionized (DI) water. 1.5 mL of the aqueous NaOH solution was added to 45.5 mL of DI water under continuous stirring in a 100 mL glass bottle. Subsequently, water‐soluble potato starch (5 g) was dissolved in the alkaline solution. Acrylonitrile (10.22 mL) was introduced dropwise using a dropping funnel. The reaction mixture was allowed to proceed for 4 h until a white, dough‐like precipitate formed. The precipitated CEStarch was collected. To purify the CEStarch, it is re‐dissolved in 20 mL of DMF and precipitated in 200 mL of DI water. The purification process was repeated three times to ensure removal of ions. Finally, the purified precipitate was collected and dried in an oven at 60°C overnight. To fabricate the SCUTE ionogels, the obtained purified CEStarch was solution‐blended with [N_1444_][DCA] using acetone/water binary solvent. SCUTE is obtained by solution casting and solvent evaporation at 60°C.

### 4.3 Material Characterization

Functional groups of the CE starch and SCUTE were identified using Vertex 80v FT‐IR spectrometer (Bruker) with a diamond ATR accessory. The spectra were obtained by averaging 64 scans at 4 cm^−1^ resolution between the wavenumber of 4000–400 cm^−1^.^1^H NMR spectra (400 MHz) were measured using JEOL JNM‐ECZL400S FT NMR system in DMSO‐d_6_. Elemental analysis (C, H, N, S) of starch and CE starch was determined using Thermo Scientific Flash EA 1112. The transmittance of SCUTE films was measured using a Shimadzu UV–Vis–NIR Spectrometer, UV‐3600, with an integrating sphere. Powder X‐ray Diffraction (XRD) patterns were collected using a Bruker D8‐Advance X‐ray diffractometer, employing Cu K*_α1_* radiation with a wavelength of 1.54056 Å. Optical microscope images were taken using Olympus BX53M. Differential scanning calorimetry (DSC) measurements were conducted using Q100 Calorimeter (TA Instruments) to determine the glass transition temperatures of the materials. Upon equilibrating at 40°C, the samples were cooled from 40°C to −80°C at a rate of 10°C/min, followed by heating to 100°C at a rate of 2°C/min. Tensile tests were conducted in accordance with ASTM D638 standard Instron 5569 universal tensile machine, with a 100 N load cell, at crosshead speed of 20 mm min^−1^. ASTM D638 type V dogbone samples with thickness of around 0.56 mm were prepared for tensile testing. A minimum of 5 specimens were tested for each sample type. For ionic conductivity measurement, CR2016 coin cells were used, with two stainless steel (SS) plates serving as blocking electrodes (SS/SCUTE‐X/SS), where X = 5, 10, 15, 20, 15, and 40. The Nyquist plots for the SCUTE polymer were obtained using AC electrochemical impedance spectroscopy (EIS) with a Biologic SP‐300 battery tester, operating over a frequency range of 10^−2^ to 10^6^ Hz and a signal amplitude of 5 mV (Figure S4). Rheology studies of dry and wet SCUTE, as well as SCUTE and PDMS inks were conducted using TA Instruments Discovery DHR‐3 rotational rheometer with an 8 mm parallel plate and 500 µm gap. The stress ramp measurements were conducted from a shear rate of 10^−3^ to 0.2 s^−1^. Step‐shear rate measurements were conducted in three steps, starting with a low shear rate (0.02 s^−1^) for 60 s, followed by a high shear rate (50 s^−1^) for 10 s, and then back to low shear rate (0.02 s^−1^) for 60 s. Frequency sweep measurements were conducted from an angular frequency of 0.1 to 600 rads^−1^ at oscillation stress of 0.01 to 1 Pa.

### 4.4 Self‐Healing Tests

To evaluate the mechanical healing ability of SCUTE. Tensile tests were performed before and after a designated healing period under ambient conditions. For water‐accelerated mechanical healing, a single drop of water was applied to the damaged area, followed by a 24‐h healing and drying period prior to testing. For the observation of underwater self‐healing by optical microscope, a SCUTE sheet was cut into two and first aligned in ambient environment, before submerged immediately in water and characterized under the optical microscope with 230x magnification. The electrical self‐healing ability of the SCUTEs was measured using an electrometer connected across a rectangular sheet of the material. The electrical resistance was measured throughout the incision, separation and re‐contacted stages of the material.

### 4.5 DFT Simulations

All calculations were carried out using the density functional theory (DFT) with the generalized Perdew–Burke–Ernzerhof (PBE) [62], and the projector augmented‐wave (PAW) pseudopotential planewave method as implemented in the VASP code [63, 64]. For the PAW pseudopotential, 2s^2^ and 2p^4^ valence electrons for O and 1s^1^ for H; for C and N were included, the *n* = 2 shell was included as valence configurations of 2s^2^2p^2^ and 2s^2^2p^3^; two cyanoethyl starch molecules with two possible configurations were put into a 3D box of 38Å × 22Å × 38Å, and four cyanoethyl starch molecules embedding two water molecules were put into a 3D box of 38Å × 50Å × 38Å. Only Γ‐centered k‐point grid was used for atomic optimization calculations. Energy convergence with respect to the plane wave cutoff was tested by varying this setting between 300 and 600 eV. Convergence to within 10 meV was achieved with a cutoff energy of 420 eV. Calculations with the van der Waals (vdW) correction were carried out by employing optPBE‐vdW functional [65]. Electrostatic potential (ESP) of the CE group was calculated using Mutiwfn code and plotted using VMD visualization program, respectively.

### 4.6 Electronic Skin

For the fabrication of the dome‐shaped capacitive e‐skin, a circular sheet of SCUTE‐20 with a diameter of 50 mm was used. Four slices were cut out at the four different quadrants. The remaining sheet was then used to form into a dome‐shape, by closing the gaps left by the four cut‐out slices. Water‐accelerated self‐healing was then applied to heal the seams of the re‐contacted edges, leading to a seamless three‐dimensional dome‐shape e‐skin. On the other hand, the digital e‐skin was formed by two strips of SCUTE20 films (20×10 mm) placed parallel next to each other with a 2 mm gap. The electrical setup for the SCUTE e‐skins to serve as the human‐machine interface is illustrated and discussed in Figure S10.

### 4.7 3D‐Printed Flexible Aquatic Circuit Board

The synthesis of underwater healable PDMS with urea linkages were similar to our previous work [61]. In brief, 1 equivalent isophorone diisocyanate (dissolved in THF) was added dropwise to a solution of poly(dimethylsiloxane), bis(3‐aminopropyl) (70%—Mn 2500; 30% Mn 27000) dissolved in THF, at room temperature. After allowing the reaction to take place for 3 h, the resultant solution was cast onto a Teflon petri dish, and dried overnight in oven at 60°C. The SCUTE40 ink was formulated at 0.5 g/ml in acetonitrile, while the PDMSurea ink was formulated at 2 g/mL in 2‐propanol, to achieve the required rheological behaviour for direct‐ink‐writing. Printing of the flexible aquatic circuit board was conducted using Cellink BIOX bioprinter. Both inks were loaded into 3 mL syringe on separate pneumatic printheads. They were printed using 25G tapered dispensing tips, with 60–80 kPa and 100–150 kPa air pressure, respectively with print speed between 5–9 mm s^−1^. The printed components were left in ambient to dry out the volatile acetonitrile and 2‐propanol solvent, respectively.

## Author Contributions

J.J.K. conceptualized the idea and designed the experiments. J.L., X.Q.K., B.H., and G.J.H.L. carried out most of the experiments. S.C.L. and J.J.W.C. designed the electronics and robotics systems for measurements and demonstrations. Y.W.Z. and Z.Y. designed and carried out the DFT computational simulations. W.T. and C.H. analysed and validated the experimental results.

## Conflicts of Interest

The authors declare no conflicts of interest.

## Supporting information




**Supporting File 1**: advs74355‐sup‐0001‐SuppMat.docx.

**Supporting File 2**: advs74355‐sup‐0002‐VideoS1.mp4.

**Supporting File 3**: advs74355‐sup‐0003‐VideoS2.mp4.

**Supporting File 4**: advs74355‐sup‐0004‐VideoS3.mp4.

**Supporting File 5**: advs74355‐sup‐0005‐VideoS4.mp4.

**Supporting File 6**: advs74355‐sup‐0006‐VideoS5.mp4.

## Data Availability

The data that support the findings of this study are available from the corresponding author upon reasonable request.
