# Supplementary material for: Self‐Healing Starch‐Based Ionogels with Hydroneutral Dipole–Dipole Interactions
Source: Adv Sci (Weinh). 2026 Feb 10;13(24):e23541. doi: 10.1002/advs.202523541 (PMC13115982; doi:10.1002/advs.202523541)
Supplement: Supplementary file 1 — Supporting File 1: advs74355‐sup‐0001‐SuppMat.docx. [file ADVS-13-e23541-s003.docx]

Supplementary Information

**Self-healing Starch-based Ionogels with Hydroneutral Dipole-dipole Interactions**

*J. Justin Koh ^a^*, Jiayi Liu ^a,b^, Xue Qi Koh ^b^,* *Binting Huang ^a,b^, Szu Cheng Lai ^a^, Jayce Jian Wei Cheng ^a^, Gwendolyn J.H. Lim ^c^,* *Warintorn Thitsartarn ^a^, Yong-Wei Zhang ^b,d^, Zhigen Yu ^c^*, Chaobin He ^a,b^**

^a^ Institute of Materials Research and Engineering (IMRE), Agency for Science, Technology and Research (A*STAR), 2 Fusionopolis Way, Innovis #08-03, Singapore 138634, Republic of Singapore

^b^ Department of Materials Science and Engineering, National University of Singapore, 9 Engineering Drive 1, Singapore 117576, Republic of Singapore

^c^ School of Materials Science and Engineering, Nanyang Technological University, 50 Nanyang Ave., Singapore 639798, Singapore

^d^ Institute of High Performance Computing (IHPC), Agency for Science, Technology and Research (A*STAR), 1 Fusionopolis Way, #16-16 Connexis, Singapore, Republic of Singapore

*****Corresponding Author:** Dr. J. Justin Koh (email: [justin_koh@a-star.edu.sg](mailto:justin_koh@a-star.edu.sg)); Prof. Zhigen Yu (email: [yuzg@a-star.edu.sg](mailto:yuzg@ihpc.a-star.edu.sg)); Prof Chaobin He (email: [msehc@nus.edu.sg](mailto:msehc@nus.edu.sg))

**Supplementary Table**

**Table S1.** Determination of CEStarch degree of substitution by elemental anlaysis.

| **Material** | **N (%)** | **DS** |
| --- | --- | --- |
| **Starch** | 0 | 0 |
| **CEStarch** | 9.75 ± 0.01 | 1.79 |

The chemical structure of pristine potato starch does not contain nitrogen element. Grafting of the cyanoethyl group introduces the nitrogen element that can be determined from elemental analysis. The amount of nitrogen determined for the CEStarch synthesized in this work is 9.75 ± 0.01 %. The degree of substitution (DS) is calculated using the following equation:

$$DS= \frac{162 \times N(\%)}{1400-53 \times N (\%)}$$

**Table S2.** Physical properties of SCUTEs and some self-healing polymeric materials in literature.

| **Tensile Modulus (MPa)** | **Tensile Strength**  **(kPa)** | **Elongation at break  (%)** | **Conductivity**  **(S cm^-1^)** | **Transparency** | **Reference** |
| --- | --- | --- | --- | --- | --- |
| 2.5 ± 0.8 | 87.7 ± 3.6 | 1414 ± 62 | 8.85 ± 0.640 ×10^-5^ | transparent | **This work**  **SCUTE-20** |
| 0.30 ± 0.02 | 11.4 ± 0.2 | 2104.2 ± 254.1 | 3.56 ± 0.093 ×10^-4^ | transparent | **This work**  **SCUTE-30** |
| Not reported | ^a^100 | ^a^1500 | ^a^10^-6^ | transparent | ^[1]^  20% [EMIM][TFSI] |
| 0.21 | ^a^80 | ^a^2000 | ^a^10^-5^ | transparent | ^[1]^  30% [EMIM][TFSI] |
| 0.015 | ^a^12.5 | 2900 | 2.6×10^-6^ | transparent | ^[2]^ |
| 0.0034 | 6.18 | 350 | 1.3×10^-4^ | yellowish | ^[3]^ |
| 0.0034 | ^a^95 | ^a^1300 | Non-conductive | yellowish | ^[4]^ |
| Not reported | ^a^170 | 2168 | ^a^8×10^-8^ | translucent | ^[5]^ |
| 0.1 | ^a^20 | ^a^1500 | 5.66×10^-5^ | transparent | ^[6]^ |
| 0.0103 | ^a^38 | ^a^900 | 1.3×10^-4^ | transparent | ^[7]^ |
| Not reported | ^a^15 | 1800 | 1.62×10^-6^ | transparent | ^[8]^ |
| Not reported | 24.6 | 650 | none | translucent | ^[9]^ |
| Not reported | 32.4 | 1410 | Conductive,  no data | transparent | ^[10]^ |
| 0.76 | ^a^135 | 580 | 5.6×10^-6^ | transparent | ^[11]^ |
| Not reported | ^a^100 | ^a^825 | ^a^10^-5^-10^-4^ | transparent | ^[12]^ |

^a^estimated from stress-strain curve or conductivity graph.

**Supplementary Table 3.** Self-healing efficiencies of SCUTEs and PFAS-based ionogels employing dipole strategy in ambient environment and in contact with water.

| **Self-healing Efficiency** | | | |
| --- | --- | --- | --- |
| **Ambient**  (24 h) | **w/ Water**  (24 h) | **Parameter of efficiency calculation** | **Reference** |
| 77.0% | 99.1% | Tensile strength | **This work**  **SCUTE-20** |
| 37.4% | 92.0% | Stretchability | **This work**  **SCUTE-20** |
| 36.1% | 28.4% | Tensile Toughness | ^[1]^  30% [EMIM][TFSI] |
| 73.7% ^a^ | 61.1% ^a^ | Tensile Strength | ^[7]^ |
| 80.1% ^a^ | 51.9% ^a^ | Stretchability | ^[7]^ |
| 61.0% | 34.0% | Tensile Toughness | ^[11]^ |
| 65.0% ^a^ | 41.4% ^a^ | Tensile Strength | ^[12]^ |
| 67.3% ^a^ | 41.2% ^a^ | Stretchability | ^[12]^ |
| ~100% | 41.8% ^a^ | Tensile Strength | ^[13]^ |
| 98.5% ^a^ | 65.6% ^a^ | Stretchability | ^[13]^ |
| 98.5% ^b^ | 77.5% ^a,b^ | Tensile Strength | ^[14]^ |
| 99.1% ^b^ | 96.4% ^b^ | Stretchability | ^[14]^ |

^a^Estimated from tensile stress-strain curve.

^b^2 h healing time.

**Supplementary Figures**

**
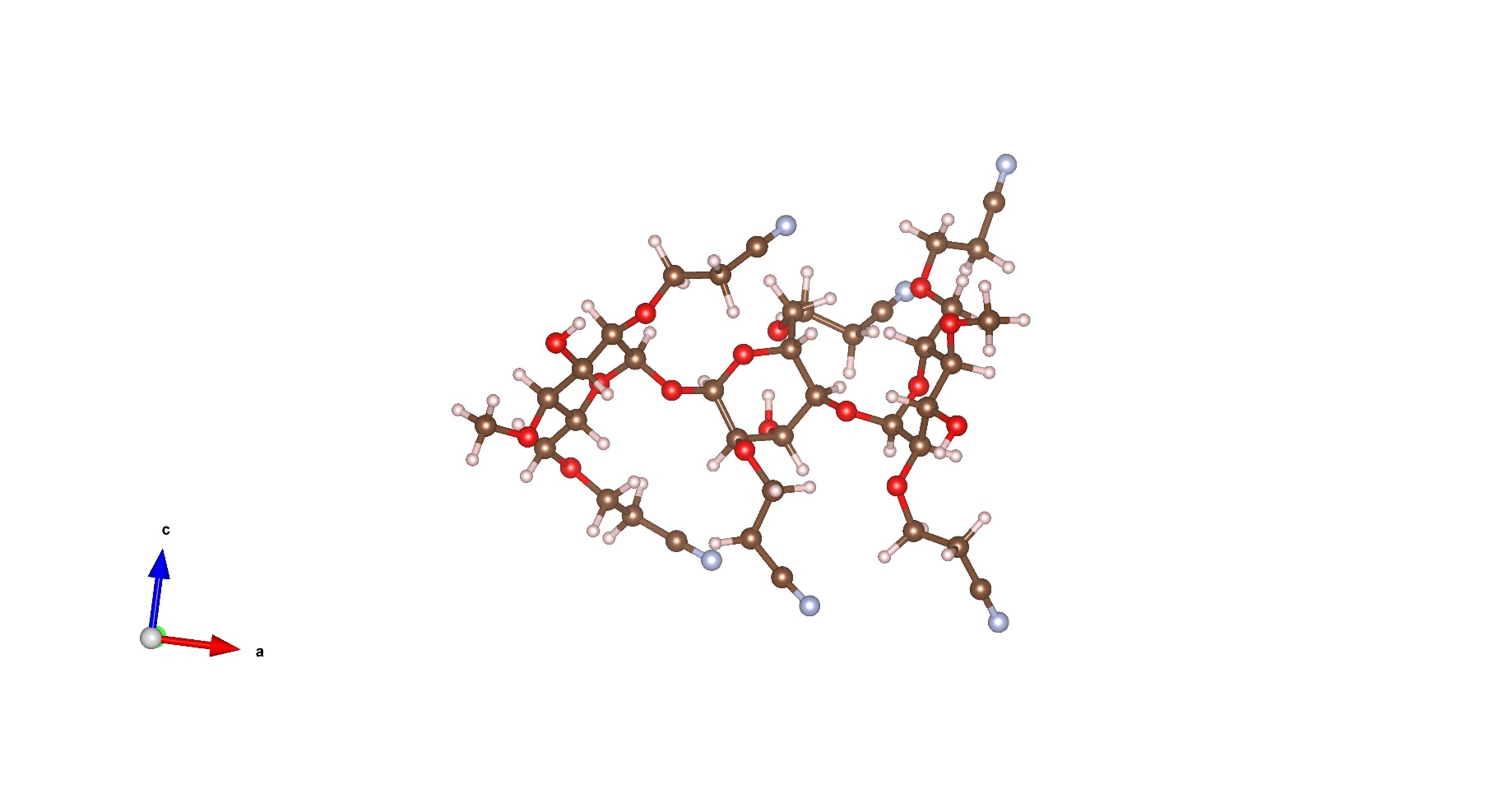
**

**Figure S1. DFT simulation model of CEStarch**

For the atomic labelling, carbon, oxygen, nitrogen and hydrogen atoms are denoted using the brow, red, blue, white spheres, respectively..

To replicate a model with similar to actual experimental results, three repeat units of glucopyranose connected by α-1,4-glycosidic linkages were constructed with the saturated terminals. Two out of three hydroxyl groups were substituted with cyanoethyl groups.


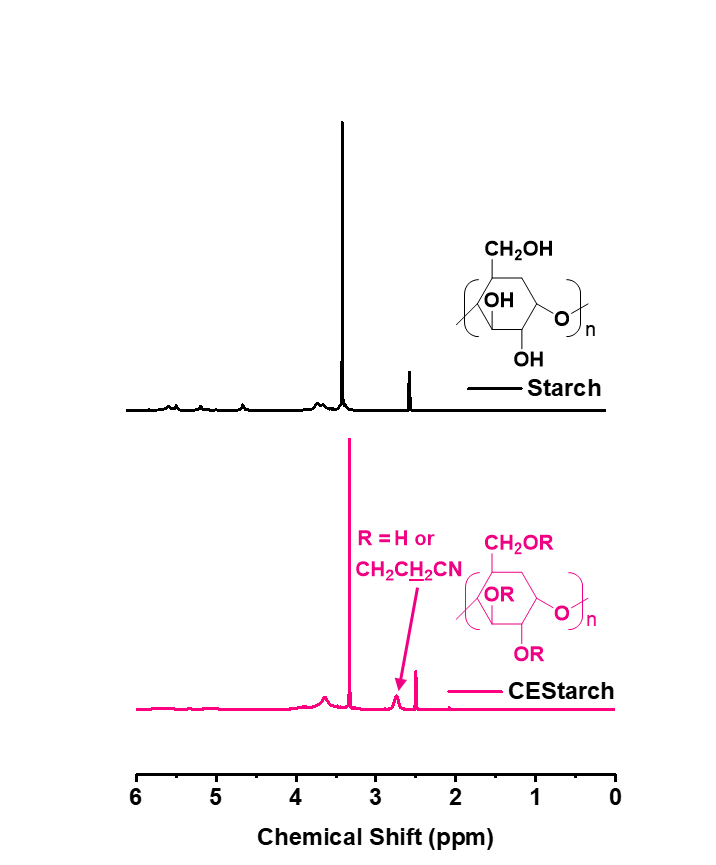


**Figure S2. ^1^H NMR spectra of pristine potato starch and CEStarch**

The distinct chemical shift peak that appears at around 2.74 ppm for CEStarch in the ^1^H-NMR spectra indicates the successfully substitution of cyanoethyl group onto Starch^[15]^.


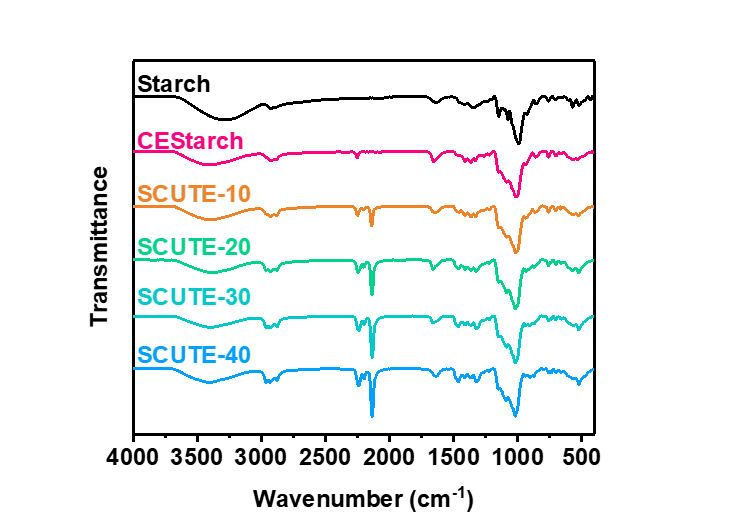


**Figure S3. ATR-FTIR spectroscopy of starch, CEStarch and SCUTEs.**


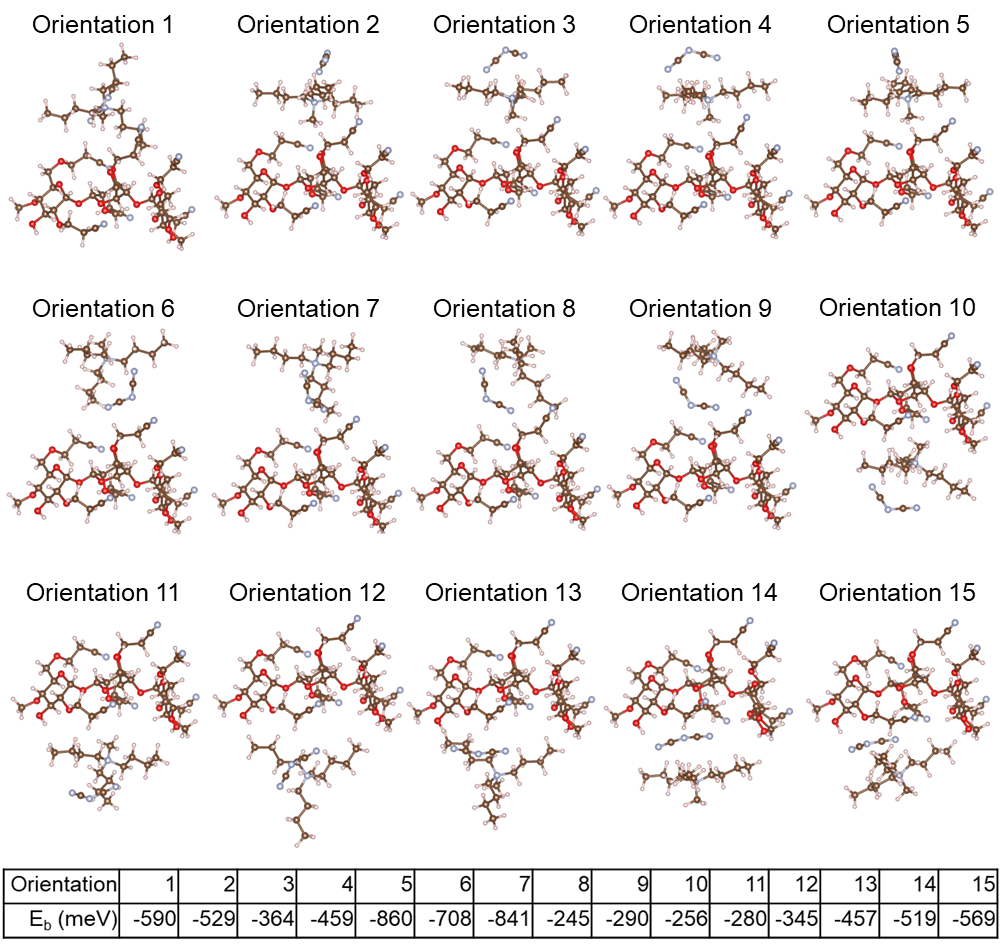


**Figure S4. Binding energy calculation through DFT simulation of** **[N_1444_][DCA] ionic liquid interacting with CE Starch at various molecular orientations.**

In general, the more negative the binding energy (E_b_), the stronger the interaction between the components. Indeed, the binding energy calculated at various molecular orientations of [N_1444_][DCA] and CEStarch are negative values of significant magnitude between 200 to 900 meV, indicating favourable interactions. This in turn suggests high compatibility between [N_1444_][DCA] and CEStarch.


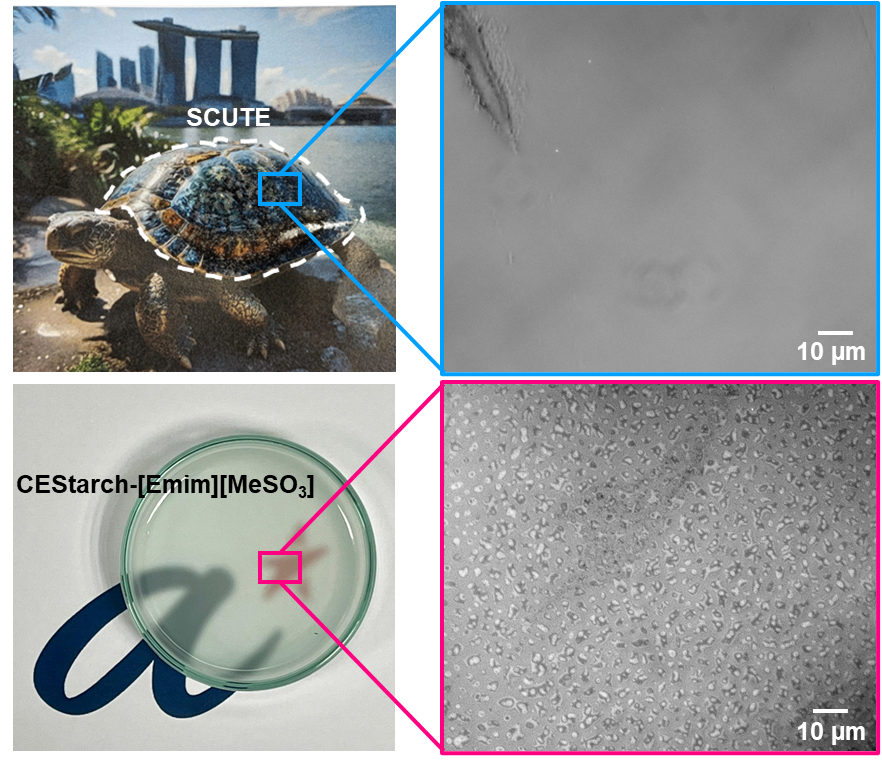


**Figure S5. Transparency and phase structure of SCUTE and CEStarch-[Emim][MeSO_3_] blends.** The incompatibility of CEStarch with [Emim][MeSO_3_] led to phase a phase separated morphology resulting in poorer optical transparency.


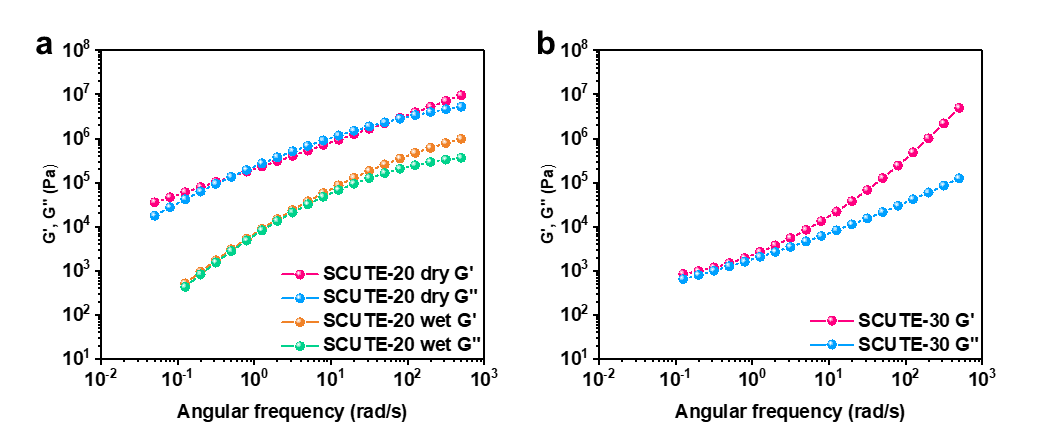


**Figure S6. Rheological oscillatory frequency sweep.** a) SCUTE-20 in dry and wet state. b) SCUTE-30 in dry state.


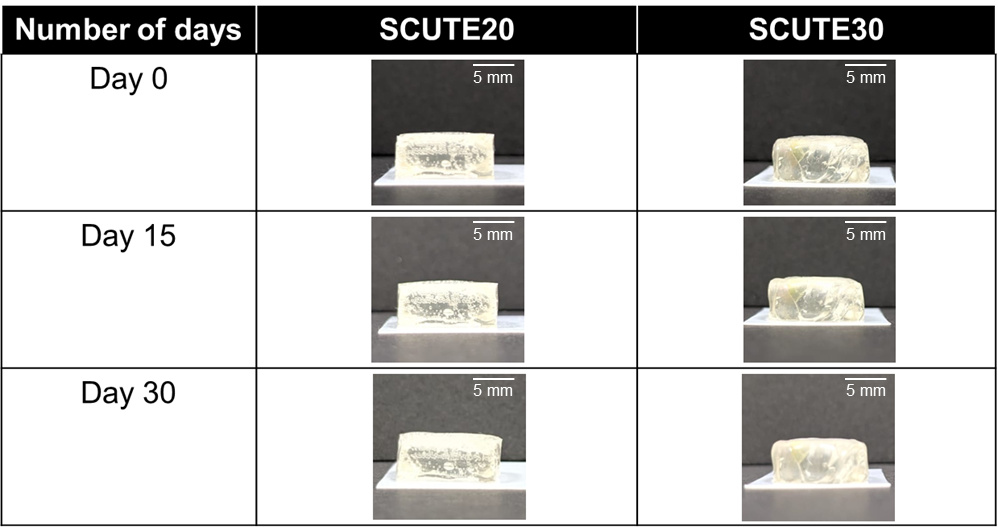


**Figure S7. Structural stability of SCUTE-20 and SCUTE-30 in ambient environment.**

**
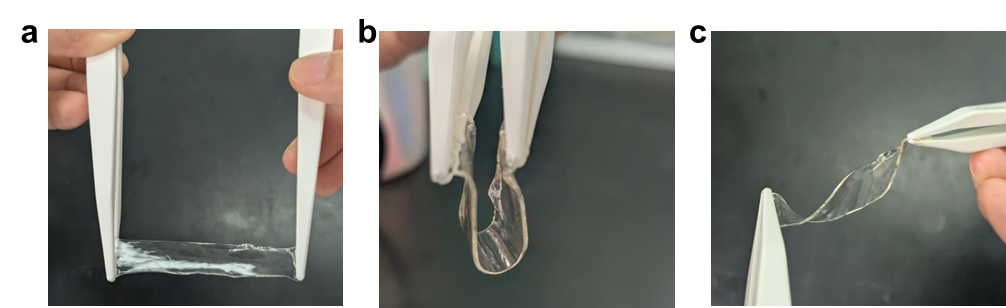
**

**Figure S8. Demonstrating easy handling of SCUTE-30.**

**
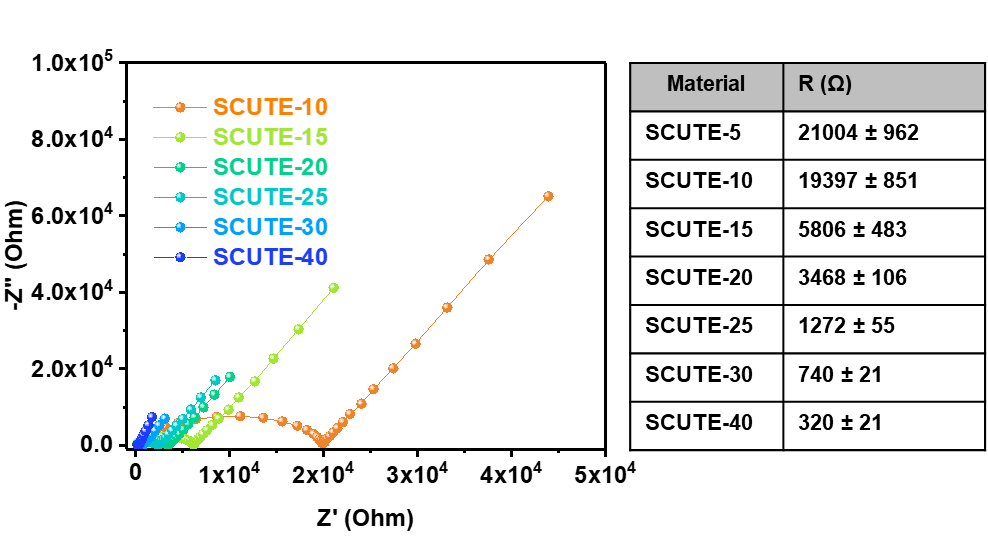
**

**Figure S9. Typical electrochemical impedance spectra of SCUTEs with different [N1444][DCA] ionic liquid content.**

The ionic conductivity (σ) of the SCUTEs was calculated from the R value using the following equation:

$$\sigma= \frac{L}{RA}$$

where L represents the thickness and A denotes the effective area of SCUTE.

Nyquist plot of SCUTE-5 is omitted better illustration purposes.

**
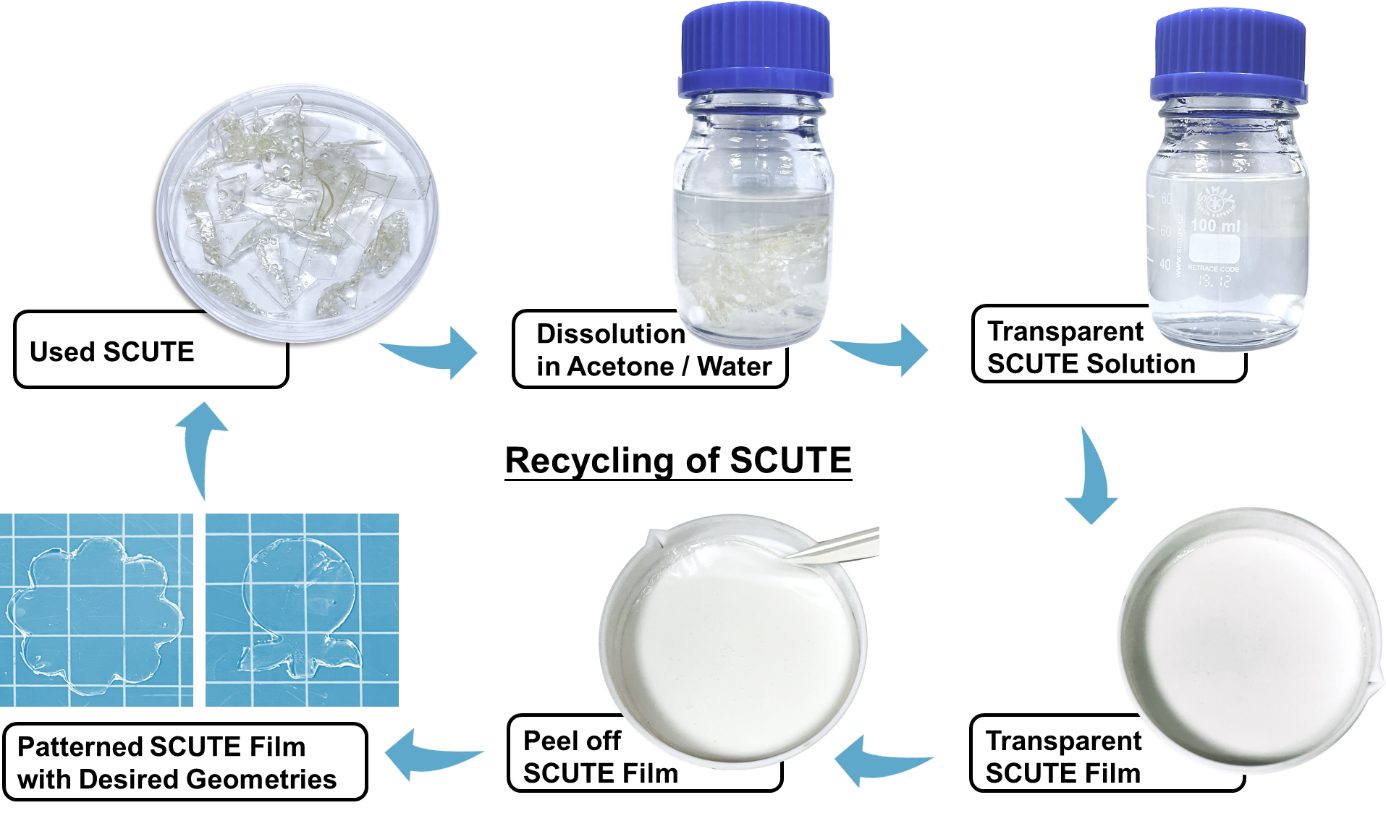
**

**Figure S10. Recycling of used SCUTE using green solvents**

**
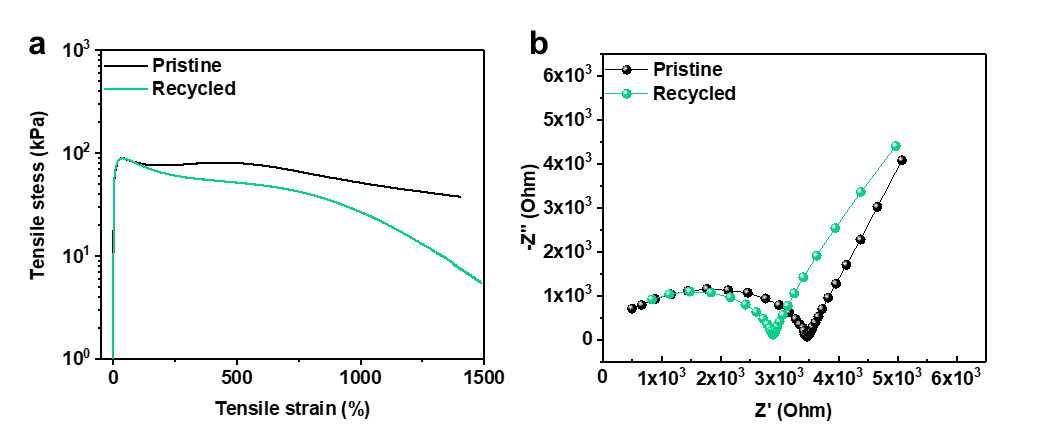
**

**Figure S11. Physical properties comparison of pristine SCUTE-20 and recycled SCUTE-20.** a) Typical tensile stress-strain curves. b) Typical electrochemical impedance spectra.

**Figure S12. Low temperature water-accelerated self-healing of SCUTE-20.** Typical tensile stress-strain curve SCUTE-20 self-healed at 4^o^C in comparison with pristine SCUTE-20.

**Figure S13. Multiple water-accelerated self-healing cycles of SCUTE-20.** Typical tensile stress-strain curve SCUTE-20 after undergoing ten cycles of water-accelerated self-healing in comparison with pristine SCUTE-20.

**
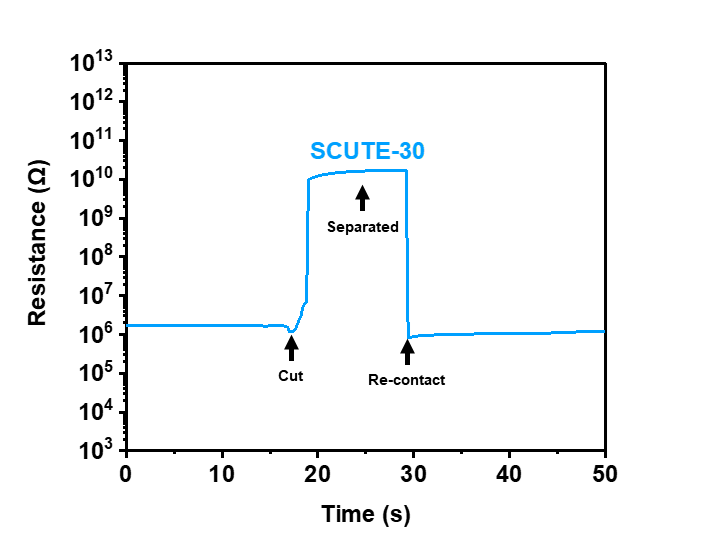
**

**Figure S14. Electrical healing of SCUTE-30.**


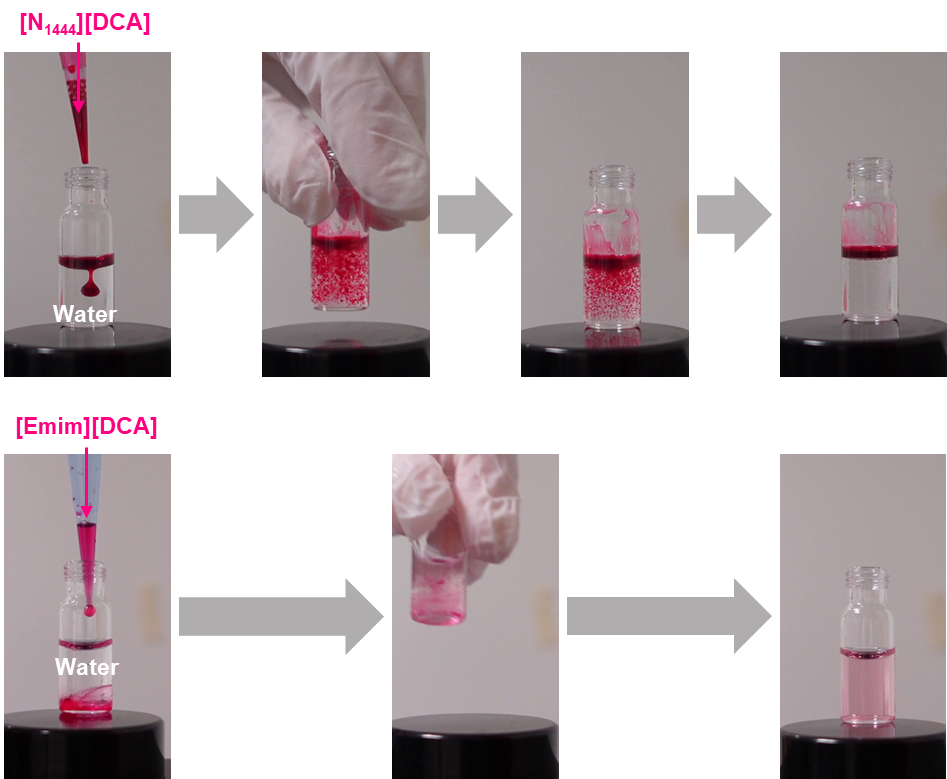


**Figure S15. Water miscibility test of ionic liquids with dicyanamide anions.** (top) [N_1444_][DCA] (bottom) [Emim][DCA].


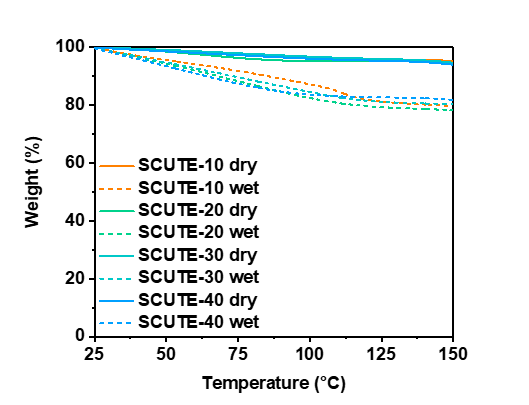


**Figure S16.** **Absorbed water content determination of wet and dry SCUTE ionogels using thermogravimetric analysis.**


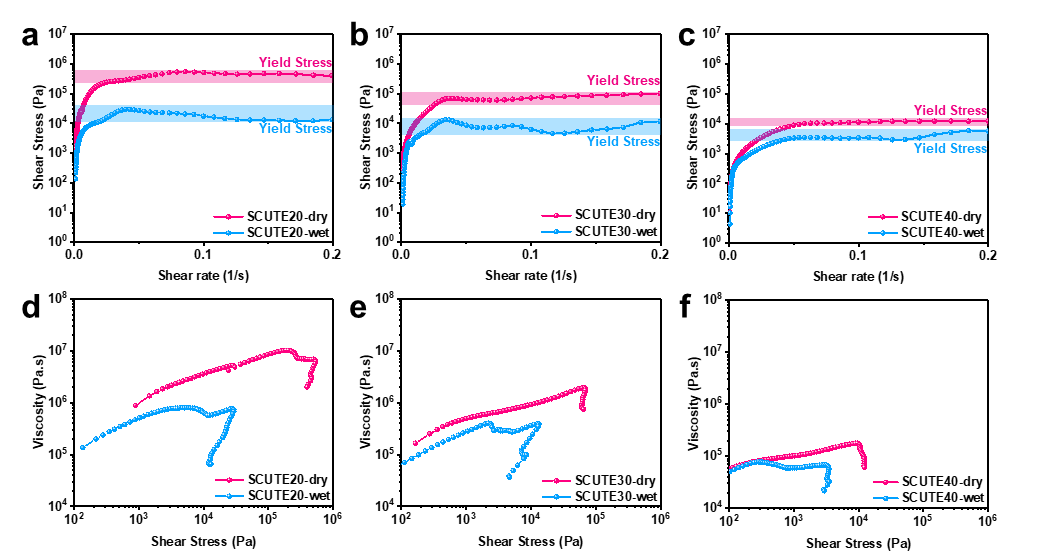


**Figure S17. Rheological stress ramp measurement. a-c** Shear stress as a function of shear rate and **d-e** Viscosity as a function of shear stress, conducted at shear rate of 10^-3^ to 0.2 s^-1^.

**
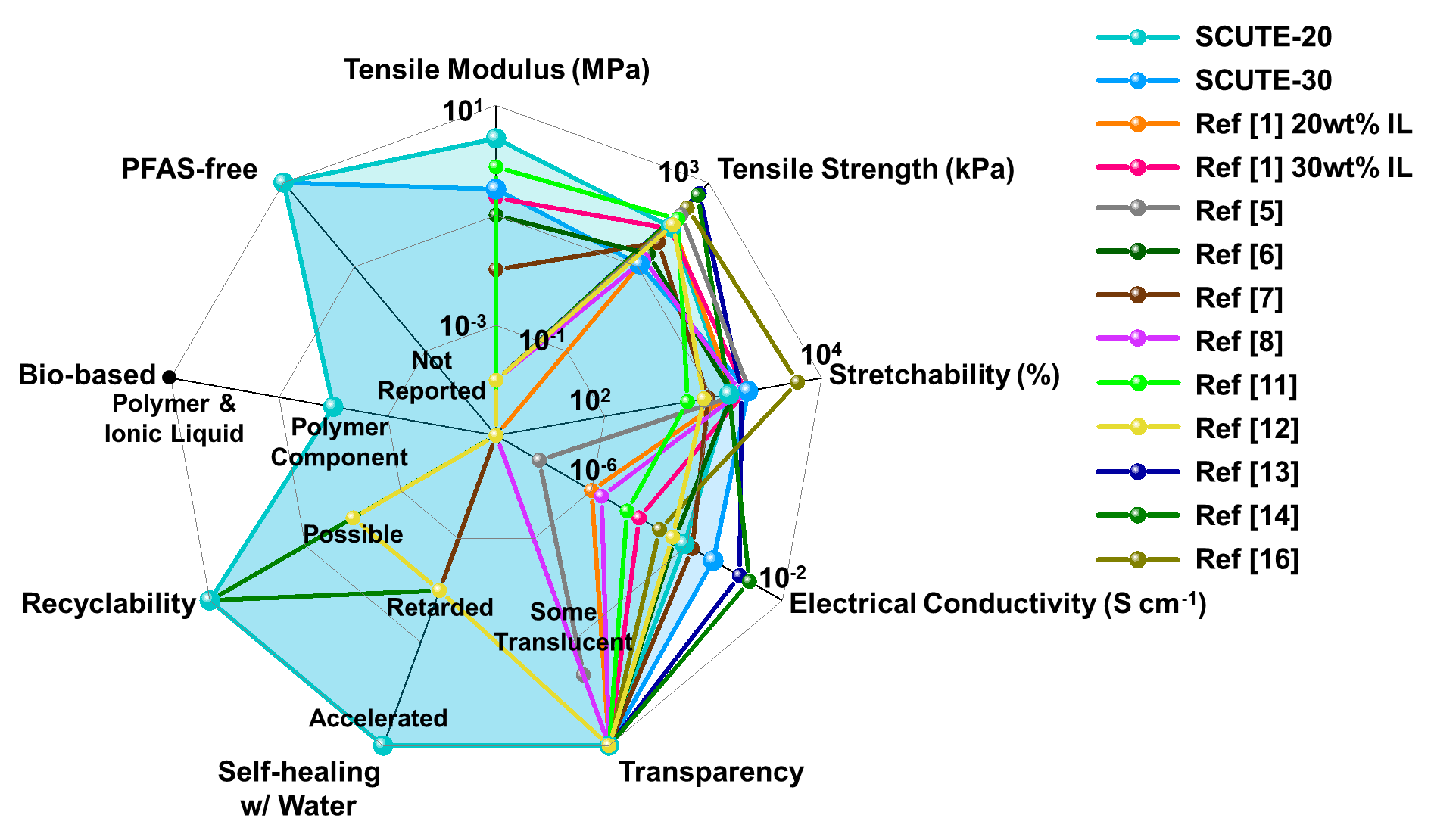
**

**Figure S18. Radar plot on key performance and characteristics of SCUTEs and ionogels in literature employing dipole strategy.** ^[1,5–8,11–14,16]^


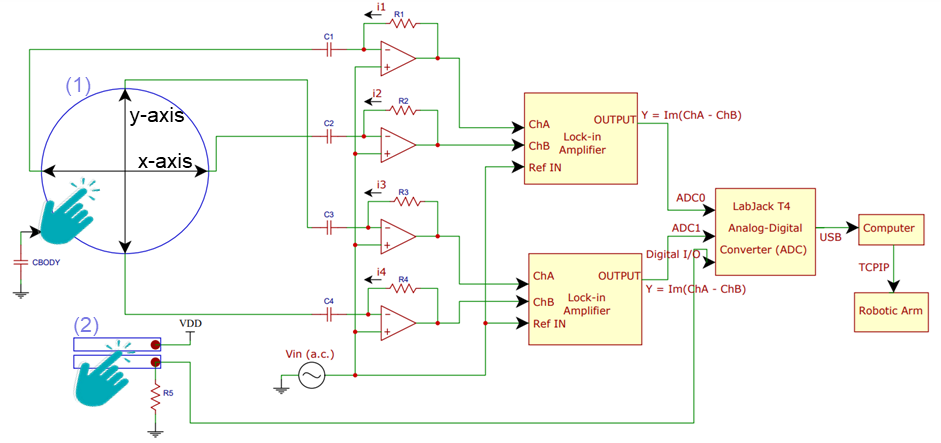


**Figure S19. Schematic illustrating electrical setup for SCUTE e-skin control of robotic arm.**

The schematic illustrates the electronic setup for a robotic arm control system using an SCUTE impedance-sensitive e-skin (1). The SCUTE dome-shaped e-skin detects finger positioning along the x- and y-axis for precise 2D tracking, operating by measuring impedance variations or current asymmetry induced by finger contact to enable accurate motion tracking and input control.

An analog front-end circuit with operational amplifiers interfaces with the touchpad, amplifying asymmetric current along the axes generated by the reference a.c. voltage source (Vin) as the signals flow into the e-skin, under the influence by the human body capacitor (CBODY) coupled via the finger. Lock-in amplifiers are used to extract and amplify the asymmetric current, with one assigned to each axis. The extracted signals are mapped to finger positions, which is then digitized by an ADC module (LabJack T4) for USB communication with a computer for controlling the robotic arm.

The system also includes a digital e-skin formed by a SCUTE electrode pair (2) in the form of rectangular strips, designed to detect finger contact for toggling the catch-release action of the robotic arm. One electrode is connected to VDD, while the other is weakly pulled to ground via a high-ohmic resistor and linked to the digital input of the LabJack T4. When a finger touches both electrodes, a high state is introduced to the digital input port, triggering the grab or release motion.

**
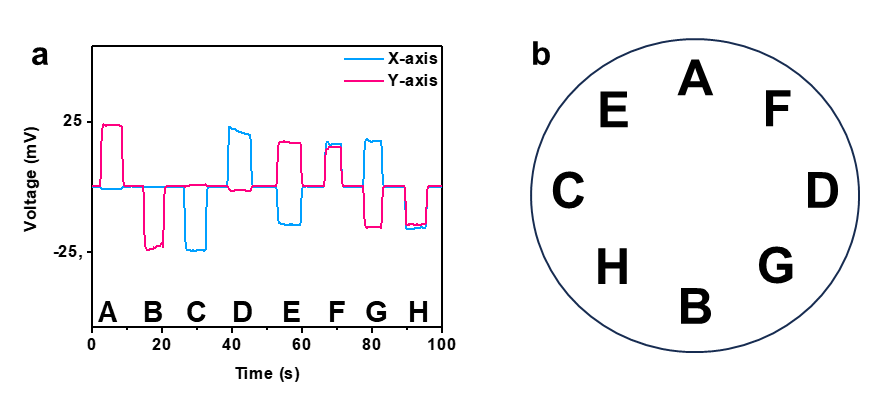
**

**Figure S20. Voltage readings of both lock-in amplifiers when various positions on dome-shaped SCUTE e-skin are touched as a function of time. a** Voltage reading of both lock-in amplifiers. **b** Position on SCUTE e-skin corresponding to the voltage readings of the lock-in amplifiers.

**
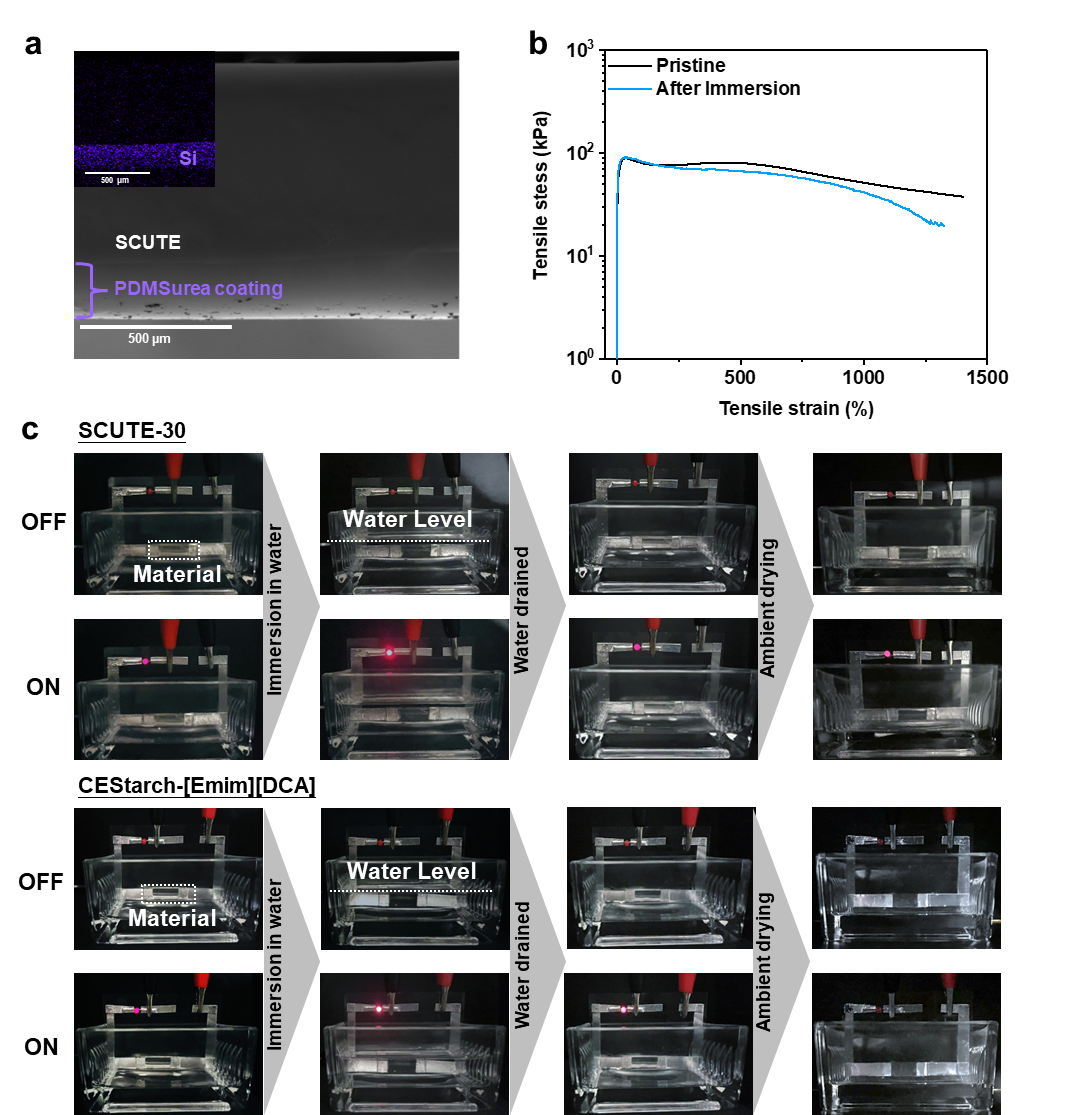
**

**Figure S21. Demonstration of SCUTEs coated with thin PDMSurea protective layer for enhanced water resistance. a** Cross-sectional scanning electron microscopy (SEM) image of cryo-fractured PDMSurea-coated SCUTE. Inset shows the energy-dispersive X-ray (EDX) mapping of Si elemental mapping from PDMS. **b** Typical stress-strain curve of pristine SCUTE-20 and coated SCUTE-20 after extended immersion in aqueous medium for 6 hours. **c** Demonstration of coated SCUTE-30 electrical conductivity retention versus CEStarch infused with water miscible [Emim][DCA].

Coating process: The thin PDMSurea coating was prepared using 0.1g/ml PDMSurea in 2-propanol and coated on SCUTEs at volume of 0.09ml/cm^2^ and dried for 15 mins in oven at 60^o^C.

Conductivity retention demonstration: Both the coated SCUTE-30 and CEStarch-[Emim][DCA] form a part of the closed circuit connected a red LED bulb, which would light up when voltage is applied. Water is then introduced to submerge the portion of the circuit that consists of the ionogels, both LED bulbs shone brighter as consequence of increased ionic conductivity of the ionogels due to water absorption. After immersion for 6 hours the water was drained and the ionogels were left to dry in ambient conditions for 24 h. Using the same initial voltage applied, the dried SCUTE-30 is able to light up the LED bulb with similar initial brightness, whereas the LED bulb in the CEStarch-[Emim][DCA] circuit remains off, indicating the loss of conductivity of the latter due to severe ion leeching into the aqueous environment due to submersion over an extended period.


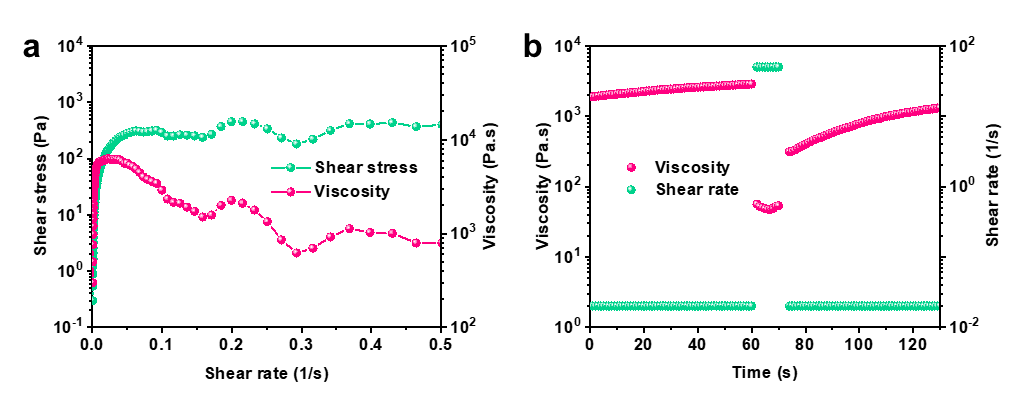


**Figure S22. Rheological behaviour of PDMS ink. a** Stress ramp method with shear stress and viscosity as a function of shear rate. **b** Step-shear-rate measurement, with viscosity as a function of three shear rate steps (low, high, low).

**References**

[1] Y. Cao, Y. J. Tan, S. Li, W. W. Lee, H. Guo, Y. Cai, C. Wang, B. C.-K. Tee, *Nat. Electron.* **2019**, *2*, 75.

[2] H. Ye, B. Wu, S. Sun, P. Wu, *Nat. Commun.* **2024**, *15*, 885.

[3] H. Wang, A. Gupta, Q. Lu, W. Wu, X. Wang, X. Huang, X. Hu, P. S. Lee, *Nat. Commun.* **2025**, *16*, 7405.

[4] H. Guo, Y. Han, W. Zhao, J. Yang, L. Zhang, *Nat. Commun.* **2020**, *11*, 2037.

[5] H. Xiang, X. Li, B. Wu, S. Sun, P. Wu, *Adv. Mater.* **2023**, *35*, 2209581.

[6] Y. Cao, T. G. Morrissey, E. Acome, S. I. Allec, B. M. Wong, C. Keplinger, C. Wang, *Adv. Mater.* **2017**, *29*, 1605099.

[7] J. Gao, E. Chen, W. Yuan, C. Meng, J. Wu, S. Guo, *Small* **2025**, *n/a*, 2502449.

[8] X. Ming, J. Du, C. Zhang, M. Zhou, G. Cheng, H. Zhu, Q. Zhang, S. Zhu, *ACS Appl. Mater. Interfaces* **2021**, *13*, 41140.

[9] W.-P. Chen, D.-Z. Hao, W.-J. Hao, X.-L. Guo, L. Jiang, *ACS Appl. Mater. Interfaces* **2018**, *10*, 1258.

[10] X. Su, H. Wang, Z. Tian, X. Duan, Z. Chai, Y. Feng, Y. Wang, Y. Fan, J. Huang, *ACS Appl. Mater. Interfaces* **2020**, *12*, 29757.

[11] Y. Zhang, M. Li, B. Qin, L. Chen, Y. Liu, X. Zhang, C. Wang, *Chem. Mater.* **2020**, *32*, 6310.

[12] L. Jia, J. Xiao, Y. Tan, K. Zhang, Y. Liu, X. Wang, *Small* **2024**, *20*, 2309231.

[13] L. Xu, Z. Huang, Z. Deng, Z. Du, T. L. Sun, Z.-H. Guo, K. Yue, *Adv. Mater.* **2021**, *33*, 2105306.

[14] Y. Zhao, F. Wang, J. Liu, D. Gan, B. Lei, J. Shao, W. Wang, Q. Wang, X. Dong, *ACS Appl. Mater. Interfaces* **2023**, *15*, 28664.

[15] E. Nakayama, J.-I. Azuma, *Cellulose* **1998**, *5*, 175.

[16] P. Shi, Y. Wang, W. W. Tjiu, C. Zhang, T. Liu, *ACS Appl. Mater. Interfaces* **2021**, *13*, 49358.
